# Comparison of Outcomes Between ST-Segment Elevation and Non-ST-Segment Elevation Myocardial Infarctions Based on Left Ventricular Ejection Fraction

**DOI:** 10.3390/jcm13226744

**Published:** 2024-11-09

**Authors:** Yong Hoon Kim, Ae-Young Her, Seung-Woon Rha, Cheol Ung Choi, Byoung Geol Choi, Soohyung Park, Su Jin Hyun, Jung Rae Cho, Min-Woong Kim, Ji Young Park, Myung Ho Jeong

**Affiliations:** 1 Division of Cardiology, Department of Internal Medicine, Kangwon National University College of Medicine, Kangwon National University School of Medicine, Chuncheon 24289, Republic of Korea; hermartha1@gmail.com; 2 Cardiovascular Center, Korea University Guro Hospital, Seoul 08308, Republic of Korea; wmagpie@korea.com (C.U.C.); shp503@naver.com (S.P.); hyunsjin62@gmail.com (S.J.H.); 3 Department of Biomedical Laboratory Science, Honam University, Gwangju 62399, Republic of Korea; trv940@naver.com; 4 Cardiology Division, Department of Internal Medicine, Kangnam Sacred Heart Hospital, Hallym University College of Medicine, Seoul 07441, Republic of Korea; jrjoe@naver.com; 5 Department of Cardiology, Changwon Hanmaeum Hospital, Hanyang University College of Medicine, Changwon 51139, Republic of Korea; kim-mw@hanmail.net; 6 Division of Cardiology, Department of Internal Medicine, Cardiovascular Center, Nowon Eulji Medical Center, Eulji University, Seoul 01830, Republic of Korea; cisamoe@gmail.com; 7 Department of Cardiovascular Center, Gwangju Veterans Hospital, Gwangju 62284, Republic of Korea; myungho@chollian.net

**Keywords:** death, drug-eluting stents, ST elevation myocardial infarction, non-ST elevation myocardial infarction

## Abstract

**Background**: This study was conducted to address the lack of reports comparing the clinical outcomes of non-ST-segment elevation myocardial infarction (NSTEMI) and STEMI based on left ventricular ejection fraction (LVEF). **Methods**: A total of 9854 patients from the Korea Acute Myocardial Infarction Registry-National Institute of Health dataset were classified into three LVEF categories: heart failure (HF) with reduced ejection fraction (EF) (HFrEF, *n* = 1250), HF with mildly reduced EF (HFmrEF, *n* = 2383), and HF with preserved EF (HFpEF, *n* = 6221). Each group was further divided into NSTEMI and STEMI groups. The primary clinical outcome was the incidence of patient-oriented composite outcomes, defined as all-cause death, recurrent myocardial infarction, any repeat coronary revascularization, hospitalization for HF, and stroke. **Results**: Following adjustment, in-hospital mortality rates were comparable between the NSTEMI and STEMI groups in the HFrEF and HFmrEF groups. However, 3-year mortality rates were higher in the NSTEMI group. In contrast, in the HFpEF group, the STEMI group had higher rates of in-hospital all-cause death (*p* = 0.001) and cardiac death (*p* < 0.001) compared to the NSTEMI group, which was associated with increased 3-year all-cause death (*p* = 0.026) and cardiac death (*p* < 0.001) in the STEMI group. When in-hospital mortality was excluded, no difference in 3-year mortality rates was observed between the NSTEMI and STEMI groups in the HFpEF group. **Conclusions**: In-hospital mortality and 3-year outcomes varied across LVEF groups. Therefore, comparing NSTEMI and STEMI based on LVEF provides valuable insights into the differences in patient outcomes.

## 1. Introduction

Myocardial infarction (MI) is classified as ST-segment elevation MI (STEMI) when patients present with new ST-segment elevations in two or more contiguous leads or new bundle branch blocks with signs of ischemic repolarization. Conversely, when ST-segment elevation is absent at presentation, the condition is typically identified as non-ST-segment elevation MI (NSTEMI) [1]. In past [2,3] and recent studies [4,5], patients with STEMI have shown a higher short-term mortality tendency, whereas those with NSTEMI have demonstrated a tendency toward higher long-term mortality. While comorbidities are more prevalent among patients with NSTEMI, they are often less likely to receive guideline-based medical treatments than their STEMI counterparts [6,7]. However, most [2,3,4,5,6,7] studies have compared outcomes in the overall patient population rather than assessing outcomes within specific subgroups. Left ventricular ejection fraction (LVEF) is a critical factor in heart failure (HF) classification, impacting prognosis, treatment response, and eligibility criteria for many clinical trials [8]. Ischemia induces structural changes in cardiomyocytes and edema within 30 min, leading to progressive myocyte death after 3 h [9]. Therefore, HF is a common complication of MI, increasing the risk of mortality by at least 3–4 times [10]. Recognizing HF in its precursor stage is also essential, as it is associated with poor outcomes, and initiating treatment at this stage could reduce mortality in patients with asymptomatic systolic left ventricular (LV) dysfunction [11]. In 2016, the European Society of Cardiology introduced HF with mid-range EF or HF with mildly reduced EF (HFmrEF) for patients with an LVEF between 41% and 49% [12]. The authors hypothesized that comparing NSTEMI and STEMI outcomes based on LVEF groups—HF with reduced EF (HFrEF), HFmrEF, and HF with preserved EF (HFpEF)—would provide more detailed insights than comparisons of the overall patient population. Additionally, no such comparisons have been reported to date. Given that newer-generation drug-eluting stents (DESs) have shown superior clinical outcomes compared to bare-metal stents (BMSs) and first-generation DESs (G1-DESs), which are now largely obsolete [13], patients who received BMSs or G1-DESs were excluded from the study. This study aimed to compare 3-year follow-up outcomes.

## 2. Methods

### 2.1. Study Population

**Figure 1:**
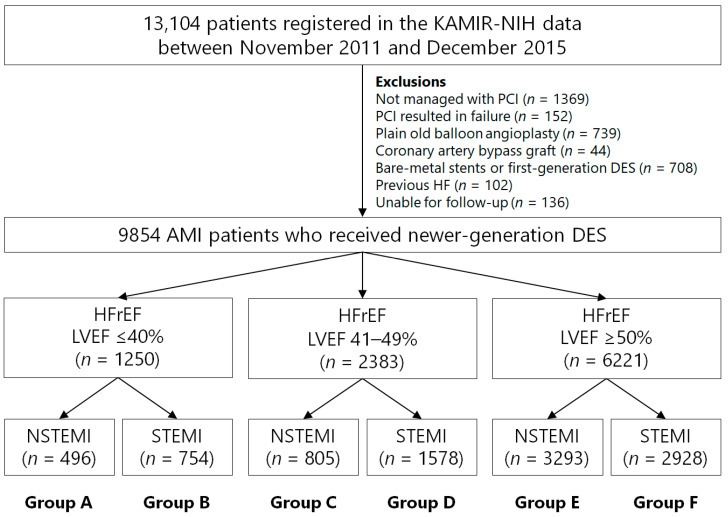
Flowchart. KAMIR-NIH, Korea Acute Myocardial Infarction Registry-National Institute of Health; PCI, percutaneous coronary intervention; DES, drug-eluting stent; HFrEF, heart failure with reduced ejection fraction; HFmrEF, heart failure with mildly reduced ejection fraction; HFpEF, heart failure with preserved ejection fraction; LVEF, left ventricular ejection fraction; NSTEMI, non-ST-segment elevation MI; STEMI, ST-segment elevation MI.

From the Korea Acute Myocardial Infarction Registry-National Institute of Health (KAMIR-NIH) [14] dataset, 13,104 patients were enrolled between November 2011 and December 2015. KAMIR-NIH is a nationwide, multicenter, prospective registry that integrates data from 20 high-volume centers in the Republic of Korea. All enrolled patients were at least 18 years old. Among the initial 13,104 patients, those who did not undergo percutaneous coronary intervention (PCI) (*n* = 1369, 10.5%) or experienced PCI failure (*n* = 152, 1.2%), those who underwent plain old balloon angioplasty (*n* = 739, 5.6%) or coronary artery bypass grafting (*n* = 44, 0.3%), those who received BMSs or G1-DESs (*n* = 708, 5.4%), those with a history of HF (*n* = 102, 0.8%), and those lost to follow-up (*n* = 136, 1.0%) were excluded (Figure 1). Ultimately, 9854 patients with AMI who successfully underwent PCI with newer-generation DESs were included. These patients were stratified by LVEF into three groups: HFrEF (*n* = 1250, 12.7%), HFmrEF (*n* = 2383, 24.2%), and HFpEF (*n* = 6221, 63.1%), and each group was further classified into STEMI and NSTEMI categories (Figure 1). The types of new-generation DESs used are listed in Table 1. During the 3-year follow-up after discharge, all patients were scheduled for evaluations at 3, 6, and 12 months, followed by assessments every 6 months. Echocardiography was advised at 12 months after AMI and annually thereafter. For patients who missed follow-up visits, outcome data were obtained through telephonic interviews or medical record reviews [15]. The Ethics Committee of each participating center, including the Korea University Guro Hospital Institutional Review Board (#KUGH MD11024), approved this non-randomized study in compliance with the 2004 Declaration of Helsinki guidelines. Written informed consent was obtained from all patients. Independent clinical research coordinators gathered data using a web-based case report form in iCReaT, a data management system by the Centers for Disease Control and Prevention, Ministry of Health and Welfare, Republic of Korea (iCReaT study number: C110016; KCT-0000863). Detailed information on the event adjudication process is available in prior publications [14]. A dedicated, impartial committee within KAMIR-NIH rigorously monitored and assessed all reported events [14].

### 2.2. PCI and Medical Treatment

According to established guidelines [16], coronary angiography and PCI were performed using a transfemoral or transradial approach. Patients scheduled for PCI received a loading dose of 200–300 mg aspirin, accompanied by either 300–600 mg clopidogrel, 180 mg ticagrelor, or 60 mg prasugrel. After PCI, all patients were advised to continue a daily regimen of 100 mg aspirin in combination with 75 mg clopidogrel, 90 mg ticagrelor twice daily, or 5–10 mg prasugrel for a minimum of 1 year. Operators had discretion over the choice of access site, revascularization strategy, and DESs.

### 2.3. Study Definitions and Clinical Outcomes

NSTEMI and STEMI classifications were based on current guidelines [1,17,18]. Given that LVEF is an essential measure for categorizing patients with HF in randomized controlled trials and observational studies, it was similarly used in this study to classify HF groups and was measured using Simpson’s biplane technique [8]. Successful PCI was defined as residual stenosis of <30% and Thrombolysis In Myocardial Infarction (TIMI) flow grade 3 in the infarct-related artery. The primary clinical outcome was the occurrence of patient-oriented composite outcomes (POCOs), which included all-cause death, recurrent MI, any repeat coronary revascularization, hospitalization for HF, and stroke. Secondary clinical outcomes included the individual components of POCO. All deaths were recorded as cardiac deaths (CDs) unless definitive evidence indicated a non-cardiac origin [19]. Recurrent MI was defined by either the resurgence of symptoms or new electrocardiographic changes, along with changes in cardiac troponin levels with at least one measurement exceeding the 99th percentile upper reference limit [1]. Periprocedural MI was not classified as a clinical outcome in this study. Revascularizations deemed clinically necessary and carried out after discharge from the initial hospitalization were classified as repeat events based on criteria defined by the Academic Research Consortium [20].

### 2.4. Statistical Analyses

Statistical analyses were performed using IBM Statistical Package for the Social Sciences, version 20 (Armonk, NY, USA). Intergroup differences in continuous variables were evaluated via unpaired *t*-tests, with results reported as the mean ± standard deviation or median (interquartile range). For comparisons across three groups, analysis of variance or the Jonckheere–Terpstra test was applied, followed by post hoc analysis using Hochberg’s or Dunnett–T3 tests for pairwise comparisons. Categorical variables were analyzed using the chi-square test or Fisher’s exact test as appropriate, with outcomes expressed as frequencies and percentages. Our study aimed to identify the variables listed in Table 1 that showed significant differences (*p* < 0.05) between the NSTEMI and STEMI groups within each LVEF category. Multicollinearity tests [20] for POCOs were performed on the variables with significant differences to confirm the absence of collinearity (Table S1). Variance inflation factor (VIF) values were analyzed to gauge multicollinearity. A VIF of >5 indicates significant multicollinearity [21], while other indicators included tolerance values of <0.1 or a condition index of >10 [22]. Variables demonstrating non-collinearity were subsequently included in the multivariate analysis. In the multivariate Cox proportional hazards regression analysis, the following variables were included: male sex, age, systolic blood pressure, diastolic blood pressure, heart rate, body mass index, Killip class II/III, cardiogenic shock, cardiopulmonary resuscitation (CPR) on admission, symptom-to-door time (SDT), door-to-balloon time (DBT), hypertension, diabetes mellitus (DM), dyslipidemia, previous MI, previous PCI, previous coronary artery bypass graft, previous stroke, current smoker status, peak creatine kinase myocardial band, peak troponin-I, hemoglobin, blood glucose, serum creatinine, triglycerides, high-density lipoprotein cholesterol, and aspirin use (Table S1). Hazard ratios (HRs) and 95% confidence intervals (CIs) were calculated using Cox proportional hazards regression models, with statistical significance defined as a two-tailed *p*-value < 0.05. The cumulative incidence of adverse events during follow-up was depicted using Kaplan–Meier estimates, with statistical significance assessed via the log-rank test. To minimize selection bias and account for potential confounders between NSTEMI and STEMI groups, propensity score matching (PSM) analysis was conducted, including all variables listed in Table 1. A 1:1 matching strategy was implemented to pair patients from each group, employing the nearest available pair-matching method with a caliper width of 0.01. The concordance statistic for the PSM analysis was 0.822.

## 3. Results

### 3.1. Baseline Characteristics

The baseline characteristics of the study population are summarized in Table 1 and Tables S2–S5. Table 1 shows that, across all three LVEF groups, patients in the NSTEMI group were older than those in the STEMI group and had a higher prevalence of cardiovascular risk factors, including hypertension, DM, history of MI, PCI, stroke, and multivessel disease, as well as a greater use of the transradial approach. Additionally, the NSTEMI group exhibited higher average serum creatinine levels and longer SDT and DBT. Conversely, the STEMI group had a higher incidence of cardiogenic shock, pre-PCI TIMI flow grade 0/1, and the use of glycoprotein IIb/IIIa inhibitors. Within the NSTEMI (Table S2) and STEMI (Table S3) groups, patients with an LVEF of ≤40%, compared to those with LVEF of 41–49% or ≥50%, showed a greater frequency of Killip class II/III, cardiogenic shock, CPR on admission, DM, previous MI, PCI, or stroke, involvement of the left main coronary artery or left anterior descending coronary arteries, multivessel disease, longer SDT and DBT, and a greater average deployed stent length. Table S4 presents the baseline characteristics of the study population before and after PSM analysis.

### 3.2. Clinical Outcomes

#### 3.2.1. In-Hospital Mortality

The in-hospital mortality rates are presented in Table 2. In the HFrEF and HFmrEF subgroups, in-hospital mortality rates were comparable between the NSTEMI and STEMI groups. However, within the HFpEF subgroup, the adjusted HRs (aHRs) for in-hospital all-cause death (0.380; 95% CI, 0.244–0.591; *p* = 0.001) and CD (0.309; 95% CI, 0.187–0.511; *p* < 0.001) were significantly lower in the NSTEMI group compared to the STEMI group.

#### 3.2.2. NSTEMI vs. STEMI

**Figure 2:**
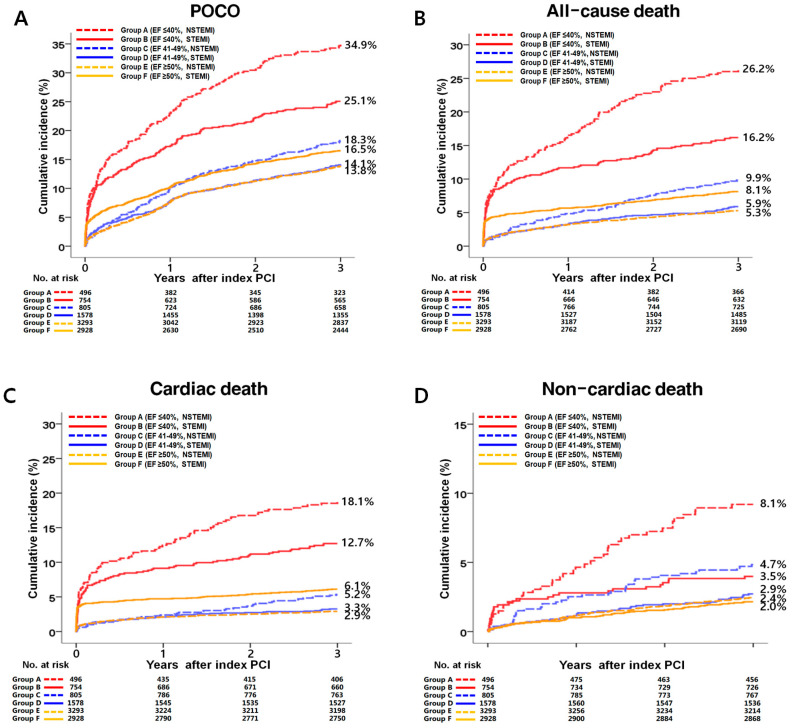

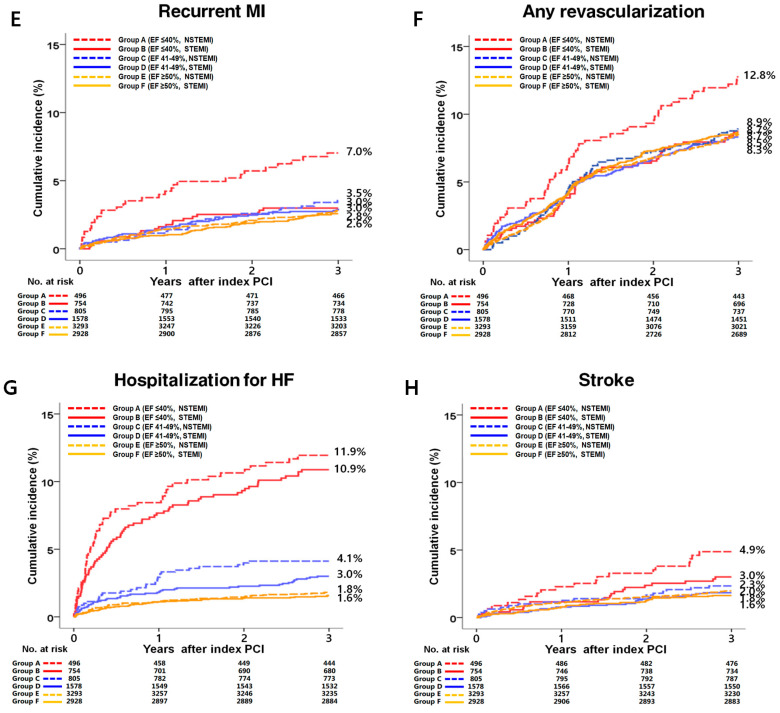
Kaplan–Meier analysis for POCO (**A**), all-cause death (**B**), cardiac death (**C**), non-cardiac death (**D**), recurrent MI (**E**), any repeat revascularization (**F**), hospitalization for HF (**G**), and stroke (**H**) during a 3-year follow-up period. POCO, patient-oriented composite outcome; EF, left ventricular ejection fraction; NSTEMI, non-ST-segment elevation myocardial infarction; STEMI, ST-segment elevation myocardial infarction; PCI, percutaneous coronary intervention; MI, myocardial infarction; HF, heart failure.

The major clinical outcomes at 3 years are detailed in Table 3 and Tables S5–S7, and Figure 2A–H. In the HFrEF group, the aHRs indicated significantly higher risks for POCOs (1.447; 95% CI, 1.161–1.757; *p* = 0.001), all-cause death (1.760; 95% CI, 1.349–2.287; *p* < 0.001), CD (1.674; 95% CI, 1.225–2.288; *p* = 0.001), non-CD (NCD, 2.046; 95% CI, 1.231–3.400; *p* = 0.006), and recurrent MI (2.407; 95% CI, 1.331–4.075; *p* = 0.004) in the NSTEMI group compared to the STEMI group. In the HFmrEF group, elevated aHRs were also observed for POCOs (1.278; 95% CI, 1.026–1.593; *p* = 0.029), all-cause death (1.699; 95% CI, 1.237–2.333; *p* = 0.001), CD (1.643; 95% CI, 1.095–2.551; *p* = 0.015), and NCD (1.769; 95% CI, 1.118–2.699; *p* = 0.012) in the NSTEMI group. In contrast, in the HFpEF group, aHRs for all-cause death (0.783; 95% CI, 0.631–0.971; *p* = 0.026) and CD (0.607; 95% CI, 0.461–0.798; *p* < 0.001) were significantly lower in the NSTEMI group (Table 3). These findings were validated through PS-adjusted analyses. Table S5 compares 3-year mortality between the STEMI and NSTEMI groups based on LVEF after excluding in-hospital mortality, where POCO (aHR, 0.988; 95% CI, 0.851–1.146; *p* = 0.869), all-cause death (aHR, 0.936; 95% CI, 0.722–1.214; *p* = 0.619), and CD (aHR, 0.940; 95% CI, 0.662–1.336; *p* = 0.730) were similar in the HFpEF group. Figure S1 provides a flowchart after the exclusion of in-hospital mortality. Figure S2 displays a Kaplan–Meier curve for the clinical outcomes in this adjusted cohort.

#### 3.2.3. HFrEF vs. HFmrEF vs. HFpEF

Table S6 compares 3-year outcomes across the three LVEF subgroups within the NSTEMI and STEMI cohorts. In the NSTEMI group, the rates of POCOs (34.9% vs. 18.3% vs. 13.8%, respectively, log-rank *p* < 0.001), all-cause death (26.2% vs. 9.9% vs. 5.3%, respectively, log-rank *p* < 0.001), CD (18.1% vs. 5.2% vs. 2.9%, respectively, log-rank *p* < 0.001), and NCD (8.1% vs. 4.7% vs. 2.4%, respectively, log-rank *p* < 0.001) were highest in the HFrEF subgroup, moderate in the HFmrEF subgroup, and lowest in the HFpEF subgroup. In the STEMI group, the rates of POCOs (14.1% vs. 16.5%, log-rank *p* = 0.023), all-cause death (5.9% vs. 8.1%, log-rank *p* = 0.005), and CD (3.3% vs. 6.1%, log-rank *p* < 0.001) were significantly lower in the HFpEF subgroup compared to those in the HFmrEF subgroup. Table S7 presents a similar comparison, excluding in-hospital mortality. Compared to Table S6, notable differences in the STEMI group were absent: POCOs (13.2% vs. 13.1%, log-rank, *p* = 0.963), all-cause death (4.9% vs. 4.4%, log-rank, *p* = 0.450), and CD (2.5% vs. 2.6%, log-rank, *p* = 0.921) rates between the HFmrEF and HFpEF subgroups showed no significant differences.

#### 3.2.4. Independent Predictors

In both the overall study population and subgroups excluding patients with in-hospital mortality, factors such as old age (≥65 years), CPR on admission, hypertension, DM, non-use of beta-blockers (BBs), renin–angiotensin system inhibitors (RASIs), and statins, and multivessel disease were significant independent predictors of POCOs and all-cause death (Tables S8 and S9). Furthermore, in the total study population, cardiogenic shock independently predicted POCOs and all-cause death, whereas pre-PCI TIMI flow grade 0/1 independently predicted POCOs and all-cause death in those excluding in-hospital mortality.

#### 3.2.5. Trends in the Use of BBs, RASIs, and Statins During the 3-Year Follow-Up Period

Table S3 presents the changes in BB, RASI, and statin use over the 3-year follow-up period after discharge. At 3 years, within the HFrEF subgroup, BB and statin use was significantly higher in the STEMI group compared to the NSTEMI group (*p* = 0.002 for both), while RASI use remained comparable between groups. In the HFmrEF subgroup, BB and statin use was higher in the STEMI group compared to the NSTEMI group (*p* = 0.005 and *p* = 0.017, respectively). However, at 3 years, RASI use was higher in the NSTEMI group compared to the STEMI group (*p* = 0.012). In the HFpEF subgroup, BB use did not differ significantly between groups; however, RASI and statin use was higher in the NSTEMI group compared to the STEMI group (*p* = 0.005 and *p* = 0.021, respectively).

## 4. Discussion

The main findings of this study are as follows. (1) In the HFrEF and HFmrEF subgroups, the in-hospital mortality rates were similar between the NSTEMI and STEMI groups; however, in the HFpEF subgroup, in-hospital all-cause death and CD were significantly lower in the NSTEMI group compared to the STEMI group. (2) Over a 3-year follow-up, the HFrEF and HFmrEF subgroups showed significantly higher rates of POCOs, all-cause death, CD, and NCD in the NSTEMI group than in the STEMI group. Conversely, in the HFpEF subgroup, 3-year all-cause death and CD rates were significantly lower in the NSTEMI group than in the STEMI group. (3) In the analysis excluding patients with in-hospital mortality, the HFrEF and HFmrEF subgroups maintained significantly higher 3-year rates of POCOs, all-cause death, CD, and NCD in the NSTEMI group compared to the STEMI group. However, in the HFpEF subgroup, POCOs, all-cause death, CD, and NCD rates were comparable between the NSTEMI and STEMI groups. (4) Across the total study population and in the subgroup excluding patients with in-hospital mortality, old age, CPR on admission, hypertension, DM, non-use of BBs, RASIs, and statins, and multivessel disease were significant independent predictors of POCOs and all-cause death. Additionally, cardiogenic shock was a significant independent predictor of POCOs and all-cause death in the total study population.

HF is a common complication of MI due to the effects of recurrent myocardial ischemia, ventricular remodeling, stunned myocardium, and hibernating myocardium on the development of LV systolic dysfunction [23]. Consequently, this study compared the long-term clinical outcomes of NSTEMI and STEMI by categorizing patients into three groups based on their LVEF (HFrEF, HFmrEF, and HFpEF) rather than analyzing the entire cohort collectively. As shown in Table 1 and Table S5, the baseline characteristics of the NSTEMI group in our study were similar to those reported in previous NSTEMI research [6,7].

In the total study population, in-hospital all-cause death (*p* = 0.009) and CD (*p* = 0.004) were significantly higher in the STEMI group than in the NSTEMI group (Table 2). However, in the HFrEF and HFmrEF subgroups, the in-hospital mortality rates were similar between the NSTEMI and STEMI groups, suggesting that these results were influenced by outcomes within the HFpEF group. Without dividing patients into three groups based on LVEF, these subgroup-specific findings would have been overlooked. Numerous studies [24,25,26,27] attribute the higher in-hospital mortality rate in patients with STEMI to a greater prevalence of cardiogenic shock. A recent study [27] reported that cardiogenic shock significantly increased the incidence of major adverse cardiac events (*p* < 0.001), all-cause death (*p* < 0.001), and CD (*p* < 0.001). In our study, the higher cardiogenic shock rate among patients with STEMI in the HFpEF group (8.0% vs. 1.6%; *p* < 0.001, Table 1) likely contributed to their elevated in-hospital mortality compared to the NSTEMI group. Additionally, previous research [27] identified higher LVEF as an independent predictor of survival (aHR, 0.967, 95% CI, 0.951–0.984, *p* < 0.001). Consistent with this, our findings demonstrated that cardiogenic shock independently predicted POCOs (aHR, 1.324; *p* = 0.002) and all-cause death (aHR, 1.519; *p* < 0.001) across the study population (Table S7).

As presented in Table 3, within the total study population and without excluding in-hospital mortality, the aHRs for all-cause death, CD, and NCD were significantly higher in the NSTEMI group than in the STEMI group for the HFrEF and HFmrEF groups. These differences remained significant even after excluding in-hospital mortality from the analysis. This finding aligns with previous studies that compared overall NSTEMI and STEMI patient populations without stratifying by LVEF [2,3,4,5]. In the HFpEF group, all-cause death (*p* = 0.021) and CD (*p* < 0.001) rates were higher in the STEMI group (Table 3). After excluding in-hospital mortality (Table S5), these rates became similar between the NSTEMI and STEMI groups. As comparative studies on NSTEMI and STEMI outcomes stratified by LVEF are lacking, our findings cannot be directly compared to those of prior studies. Future studies are needed to validate these results.

In the NSTEMI group, the 3-year rates of POCOs, all-cause death, CD, and NCD were highest in the HFrEF subgroup, moderate in the HFmrEF subgroup, and lowest in the HFpEF subgroup (Tables S6 and S7). These trends might be associated with the poorer baseline characteristics observed in the HFrEF subgroup compared to the HFmrEF and HFpEF subgroups (Table S2). Our results align with recent findings [28], where reduced EF was associated with a higher risk of mortality compared to normal EF (aHR, 1.64; 95% CI, 1.36–1.96; *p* < 0.001). Additionally, mildly reduced EF was similarly associated with elevated mortality risk compared to normal EF (aHR, 1.33; 95% CI, 1.05–1.68; *p* = 0.019). In the STEMI group, before excluding in-hospital mortality, rates of POCOs, all-cause death, and CD were higher in the HFpEF subgroup than in the HFmrEF subgroup (Table S6). However, after excluding in-hospital mortality, the STEMI group showed comparable 3-year POCO, all-cause death, and CD rates across the HFmrEF and HFpEF subgroups (Table S7). This suggests that high in-hospital mortality in the STEMI group within the HFpEF subgroup contributed to the elevated 3-year POCOs, all-cause death, and CD rates (Table S6). It remains unclear whether the natural progression of HFmrEF following STEMI aligns more closely with HFrEF or HFpEF [29]. In a single-center registry [30] involving 1260 patients with STEMI, the mortality rates between the HFmrEF and HFpEF subgroups were similar (4.3% vs. 3.5%, respectively; *p* = 0.897), consistent with our findings.

As shown in Figure S3, the number of BB and statin users was greater in the STEMI group compared to the NSTEMI group within the HFrEF and HFmrEF subgroups. This finding is consistent with those of previous studies indicating that patients with NSTEMI often receive relatively less guideline-directed medical therapy compared to patients with STEMI [6,7]. In Tables S8 and S9, BB and statin use were identified as independent predictors of POCOs and all-cause death, prompting their inclusion in the multivariable-adjusted analysis collinearity test (Table S1). Despite the higher BB and statin use, the NSTEMI group exhibited a higher mortality rate than the STEMI group within the HFrEF and HFmrEF subgroups (Table 3). This difference is likely attributable to the greater burden of comorbidities in patients with NSTEMI, indicating a poorer baseline profile compared to patients with STEMI [6,7]. Notably, a recent study examining the “smoker’s paradox” [31] in 2546 patients with STEMI who underwent primary PCI and were followed for 1 year found that smokers were, on average, approximately 10 years younger than non-smokers and had significantly shorter ischemic (*p* = 0.002) and decision (*p* = 0.0063) times, along with a significantly lower mortality rate (HR, 0.54; *p* < 0.001). The authors [31] suggested that the “smoker’s paradox” could be partly explained by the younger demographics of these patients, although additional factors may also contribute.

To optimize treatment strategies and improve patient care, stratified analyses by sex were conducted (Figures S4 and S5, Tables S10–S15). In both male and female groups, the in-hospital mortality rates in the HFrEF and HFmrEF subgroups were comparable, similar to the overall study population (Table 2). However, within the HFpEF subgroup, the NSTEMI group showed lower rates of in-hospital all-cause death and CD than the STEMI group (Tables S10 and S11). During the 3-year follow-up period, consistent with the overall study population (Table 3), the male NSTEMI group exhibited higher rates of 3-year all-cause death, CD, and NCD than the STEMI group in the HFrEF and HFmrEF subgroups. Conversely, within the HFpEF subgroup, the NSTEMI group had lower rates of 3-year all-cause death and CD compared to the STEMI group (Table S12). After excluding in-hospital mortality, the 3-year mortality rates in the HFpEF subgroup were comparable between the NSTEMI and STEMI groups (Table S14), consistent with the overall findings (Table S5). For the female group, the 3-year mortality rates were similar between the NSTEMI and STEMI groups in the HFrEF and HFmrEF subgroups. In the HFpEF subgroup, the NSTEMI group showed lower rates of 3-year all-cause death and CD compared to the STEMI group. This discrepancy might be associated with the higher in-hospital mortality rate observed among females. However, when excluding in-hospital mortality, the 3-year mortality rates in the HFpEF subgroup were comparable between the NSTEMI and STEMI groups (Table S15). Our results align with the findings by Rodríguez–Padial et al. [32], who reported higher mortality in women with STEMI (odds ratio, 1.18; *p* < 0.001) and lower mortality in women with NSTEMI (odds ratio, 0.85; *p* < 0.001). Pancholy et al. [33] reported that women generally experience higher mortality from STEMI than men, potentially due to multiple factors, including higher baseline ages, less favorable cardiovascular risk factors, longer reperfusion times, fewer guideline-based treatments, and distinct STEMI pathophysiology. While our study also indicated higher mortality in females, further research with a larger patient population is warranted to validate these findings.

To our knowledge, this is the first study to compare the long-term outcomes of NSTEMI and STEMI across three LVEF classifications. Our findings indicate that the outcomes of STEMI and NSTEMI differ based on the LVEF degree. Additionally, this study demonstrates that the STEMI group had higher in-hospital mortality than the NSTEMI group and that long-term outcomes could vary depending on whether in-hospital mortality was included or excluded.

While the small sample sizes of certain NSTEMI or STEMI subgroups limit the ability to draw definitive conclusions, this study leverages data from a registry of 20 high-volume tertiary university hospitals in the Republic of Korea. It is believed that our findings will provide valuable insights for comparing the long-term outcomes of NSTEMI and STEMI.

This study has certain inherent limitations. First, as the KAMIR-NIH is a registry dataset, it might contain instances of underreported or missing data. Second, although multivariate- and propensity score-adjusted analyses were performed to enhance result accuracy, the influence of unmeasured variables not included in the registry cannot be entirely ruled out. Third, the 3-year follow-up period might be insufficient to fully capture long-term clinical outcomes. Fourth, as this study included patients enrolled between November 2011 and December 2015, advancements in HF management—such as the introduction of angiotensin receptor–neprilysin inhibitors (ARNIs) and sodium–glucose co-transporter 2 (SGLT-2) inhibitors [34]—are not reflected in our findings. These therapies are now considered essential for HFrEF management, representing a key limitation of this study [34]. Finally, changes in LVEF over the 3-year follow-up are critical to determining clinical outcomes. However, due to extensive missing values in the registry, data on LVEF changes over the 3-year follow-up period for each group cannot be provided.

## 5. Conclusions

In conclusion, the in-hospital mortality and 3-year outcomes varied across the LVEF groups. Therefore, comparing outcomes based on LVEF subgroups likely provides a more detailed understanding of the differences between NSTEMI and STEMI than comparisons between the entire NSTEMI and STEMI populations. Furthermore, it was observed that long-term outcomes might vary depending on whether in-hospital mortality is included in the analysis. Further studies are required to validate these findings.

## Figures and Tables

**Table 1:** Baseline characteristics.

	HFrEF (LVEF ≤ 40%, *n* = 1250)			HFmrEF (LVEF 41–49%, *n* = 2383)			HFpEF (LVEF ≥ 50%, *n* = 6221)		
Variables	NSTEMI (*n* = 496) Group A	STEMI (*n* = 754) Group B	*p*	NSTEMI (*n* = 805) Group C	STEMI (*n* = 1578) Group D	*p*	NSTEMI (*n* = 3293) Group E	STEMI (*n* = 2928) Group F	*p*
Male, *n* (%)	326 (65.7)	567 (75.2)	<0.001	557 (69.2)	1232 (78.1)	<0.001	2452 (74.5)	2346 (80.1)	<0.001
Age, years	69.1 ± 11.1	64.9 ± 12.8	<0.001	66.5 ± 11.7	62.3 ± 12.4	<0.001	63.0 ± 12.0	61.6 ± 12.5	<0.001
SBP, mmHg	131.9 ± 27.6	124.6 ± 27.8	<0.001	131.8 ± 25.3	129.6 ± 26.8	0.052	137.1 ± 25.9	127.8 ± 28.2	<0.001
DBP, mmHg	79.9 ± 16.7	78.1 ± 17.9	0.080	79.5 ± 15.7	79.2 ± 16.5	0.629	82.0 ± 15.1	77.8 ± 17.6	<0.001
Heart rate, beats/min	92.4 ± 20.0	86.3 ± 20.3	<0.001	80.5 ± 17.6	78.6 ± 18.1	0.015	81.9 ± 15.1	74.2 ± 18.9	<0.001
Body mass index, kg/m^2^	23.4 ± 3.8	23.5 ± 3.3	0.436	23.7 ± 3.2	24.0 ± 3.3	0.013	24.3 ± 3.3	24.3 ± 3.1	0.724
LVEF, %	32.7 ± 6.3	33.5 ± 6.1	0.027	45.8 ± 2.5	45.6 ± 2.4	0.039	59.5 ± 6.2	57.6 ± 5.8	<0.001
Killip class II/III, *n* (%)	229 (46.2)	187 (24.8)	<0.001	158 (19.6)	243 (15.4)	0.011	323 (9.8)	275 (9.4)	0.605
Cardiogenic shock, *n* (%)	18 (3.6)	61 (8.1)	0.001	14 (1.7)	81 (5.1)	<0.001	52 (1.6)	233 (8.0)	<0.001
CPR on admission, *n* (%)	59 (11.9)	111 (14.7)	0.177	29 (3.6)	75 (4.8)	0.205	54 (1.6)	187 (6.4)	<0.001
SDT, hours	11.1 (3.3–48.0)	3.1 (1.5–9.9)	<0.001	7.6 (2.7–25.8)	2.8 (1.2–6.5)	<0.001	6.1 (2.2–24.0)	2.0 (1.0–4.6)	<0.001
DBT, hours	15.5 (3.6–39.5)	0.9 (0.8–1.3)	<0.001	13.0 (3.4–24.2)	1.0 (0.8–1.3)	<0.001	13.7 (4.0–24.4)	0.9 (0.8–1.3)	0.001
Risk factors									
Hypertension, *n* (%)	305 (61.5)	349 (46.3)	<0.001	405 (50.3)	707 (44.8)	0.012	1730 (52.5)	1355 (46.3)	<0.001
Diabetes mellitus, *n* (%)	238 (48.0)	246 (32.6)	0.019	262 (32.5)	379 (24.0)	<0.001	917 (27.8)	658 (22.5)	<0.001
Dyslipidemia, *n* (%)	43 (8.7)	75 (9.9)	0.490	67 (8.3)	160 (10.1)	0.161	447 (13.6)	318 (10.9)	0.001
Previous MI, *n* (%)	69 (13.9)	59 (7.8)	0.001	69 (8.6)	82 (5.2)	0.002	172 (5.2)	99 (3.4)	<0.001
Previous PCI, *n* (%)	81 (16.3)	72 (9.5)	<0.001	85 (10.6)	97 (6.1)	<0.001	285 (8.7)	151 (5.2)	<0.001
Previous CABG, *n* (%)	11 (2.2)	4 (0.5)	0.014	8 (1.0)	6 (0.4)	0.087	16 (0.5)	8 (0.3)	0.220
Previous stroke, *n* (%)	59 (11.9)	51 (6,8)	0.002	56 (7.0)	63 (4.0)	0.003	168 (5.1)	105 (3.6)	0.004
Current smokers, *n* (%)	135 (27.2)	306 (40.6)	<0.001	271 (33.7)	732 (46.4)	<0.001	1248 (37.9)	1342 (45.8)	<0.001
Laboratory results									
Peak CK-MB, mg/dL	21.4 (6.4–68.3)	156.5 (26.0–300.0)	<0.001	35.0 (8.4–125.8)	172.0 (50.4–311.5)	<0.001	18.6 (5.5–70.5)	104.3 (29.4–224.1)	<0.001
Peak troponin-I, ng/mL	8.9 (2.5–31.5)	54.4 (16.3–168.8)	<0.001	10.8 (2.5–31.8)	47.7 (20.6–111.1)	<0.001	6.0 (1.3–21.5)	27.9 (11.5–71.9)	<0.001
Hemoglobin, mg/dL	12.4 ± 2.4	13.8 ± 2.0	<0.001	13.4 ± 1.9	14.3 ± 2.0	<0.001	13.9 ± 1.9	14.3 ± 1.9	<0.001
Blood glucose, mg/dL	197.7 ± 86.3	199.1 ± 96.1	0.828	164.3 ± 84.2	175.8 ± 75.8	0.001	152.3 ± 69.7	173.6 ± 76.3	<0.001
Serum creatinine, mg/dL	1.75 ± 0.88	1.12 ± 0.79	<0.001	1.17 ± 0.88	1.01 ± 0.83	0.002	1.05 ± 0.75	1.00 ± 0.63	0.030
Total cholesterol, mg/dL	169.0 ± 45.6	176.5 ± 46.7	0.006	176.6 ± 46.7	185.5 ± 44.1	<0.001	181.5 ± 44.3	182.6 ± 43.8	0.318
Triglyceride, mg/dL	113.1 ± 98.6	120.7 ± 94.9	0.129	121.3 ± 90.4	134.9 ± 88.8	0.001	138.0 ± 79.4	147.9 ± 82.3	0.003
HDL-cholesterol, mg/dL	42.3 ± 12.8	43.9 ± 14.6	0.055	42.5 ± 11.8	43.5 ± 11.9	0.059	42.8 ± 11.4	41.9 ± 11.7	0.001
LDL-cholesterol, mg/dL	103.9 ± 39.5	111.5 ± 44.5	0.004	110.9 ± 39.5	117.7 ± 38.8	<0.001	115.2 ± 38.3	115.8 ± 38.2	0.568
Discharge medications									
Aspirin, n (%)	483 (97.4)	729 (96.7)	0.614	796 (98.9)	1565 (99.2)	0.051	3254 (98.8)	2862 (97.7)	0.001
Clopidogrel, *n* (%)	395 (79.6)	558 (74.0)	0.029	607 (75.4)	1062 (67.4)	<0.001	2328 (70.7)	1944 (66.4)	<0.001
Ticagrelor, *n* (%)	71 (14.3)	136 (18.0)	0.087	135 (16.8)	314 (19.9)	0.068	641 (19.5)	620 (21.2)	0.100
Prasugrel, *n* (%)	30 (6.0)	60 (8.0)	0.220	63 (7.8)	202 (12.8)	<0.001	324 (9.8)	364 (12.4)	0.001
Beta-blocker, *n* (%)	395 (79.6)	595 (78.9)	0.776	681 (84.6)	1407 (89.2)	0.002	2803 (85.1)	2509 (85.7)	0.541
RASI, *n* (%)	389 (78.4)	566 (75.1)	0.174	642 (79.8)	1298 (82.3)	0.148	2732 (83.0)	2308 (78.8)	<0.001
Statin, *n* (%)	434 (87.5)	663 (87.9)	0.860	761 (94.5)	1489 (94.4)	0.925	3133 (95.1)	2712 (92.6)	<0.001
Anticoagulant, *n* (%)	36 (7.3)	47 (6.2)	0.488	21 (2.6)	53 (3.4)	0.382	30 (0.9)	44 (1.5)	0.035
Infarct-related artery									
Left main, *n* (%)	26 (5.2)	32 (4.2)	0.413	20 (2.5)	18 (1.1)	0.016	97 (2.9)	25 (0.9)	<0.001
LAD, *n* (%)	257 (51.8)	549 (72.8)	<0.001	413 (51.3)	1028 (65.1)	<0.001	1295 (39.3)	1165 (39.8)	0.716
LCx, *n* (%)	78 (15.7)	30 (4.0)	<0.001	181 (22.5)	127 (8.0)	<0.001	909 (27.6)	300 (10.2)	<0.001
RCA, *n* (%)	135 (27.2)	143 (19.0)	0.001	191 (23.7)	405 (25.7)	0.317	992 (30.1)	1438 (49.1)	<0.001
Treated vessel									
Left main, *n* (%)	36 (7.3)	39 (5.2)	0.144	38 (4.7)	32 (2.0)	<0.001	146 (4.4)	44 (1.5)	<0.001
LAD, *n* (%)	352 (71.0)	620 (82.2)	<0.001	520 (64.6)	1127 (71.4)	0.001	1786 (54.2)	1455 (49.7)	<0.001
LCx, *n* (%)	170 (34.3)	118 (15.6)	<0.001	287 (35.7)	254 (16.1)	<0.001	1320 (40.1)	536 (18.3)	<0.001
RCA, *n* (%)	195 (39.3)	203 (26.9)	<0.001	272 (33.8)	497 (31.5)	0.266	1268 (38.5)	1568 (53.6)	<0.001
Multivessel disease, *n* (%)	351 (70.8)	423 (56.1)	<0.001	475 (59.0)	733 (46.5)	<0.001	1744 (53.0)	1342 (45.8)	<0.001
Transradial approach, *n* (%)	208 (41.9)	185 (24.5)	<0.001	378 (47.0)	444 (28.1)	<0.001	1775 (53.9)	739 (25.2)	<0.001
Pre-PCI TIMI flow grade 0/1, *n* (%)	203 (40.9)	580 (76.9)	<0.001	382 (47.5)	1209 (76.6)	<0.001	1211 (36.8)	2107 (72.0)	<0.001
ACC/AHA type B2/C lesion, *n* (%)	434 (87.5)	683 (90.6)	0.092	707 (87.8)	1399 (88.7)	0.544	2742 (83.8)	2577 (88.0)	<0.001
Glycoprotein IIb/IIIa inhibitor, *n* (%)	35 (7.1)	130 (17.2)	<0.001	75 (9.3)	322 (20.4)	<0.001	294 (8.9)	651 (22.2)	<0.001
IVUS/OCT, *n* (%)	92 (18.5)	133 (17.6)	0.707	208 (25.8)	316 (20.0)	0.001	869 (26.4)	597 (20.4)	<0.001
FFR, *n* (%)	6 (1.2)	5 (0.7)	0.361	13 (1.6)	9 (0.6)	0.021	86 (2.6)	24 (0.8)	<0.001
Drug-eluting stents ^a^									
ZES, *n* (%)	118 (23.8)	170 (22.5)	0.631	190 (23.6)	363 (23.0)	0.758	773 (23.5)	679 (23.2)	0.810
EES, *n* (%)	289 (58.3)	382 (50.7)	0.009	430 (53.4)	819 (51.9)	0.488	1667 (50.6)	1446 (49.4)	0.334
BES, *n* (%)	68 (13.7)	136 (18.0)	0.050	152 (18.9)	283 (17.9)	0.575	700 (21.3)	595 (20.3)	0.381
Others, *n* (%)	21 (4.2)	66 (8.8)	0.002	33 (4.1)	113 (7.2)	0.003	153 (4.6)	208 (7.1)	<0.001
Stent diameter, mm	3.06 ± 0.41	3.12 ± 0.40	0.007	3.05 ± 0.39	3.17 ± 0.40	<0.001	3.08 ± 0.43	3.18 ± 0.42	<0.001
Stent length, mm	31.8 ± 15.4	30.9± 13.1	0.276	31.1 ± 14.9	29.3 ± 12.4	0.003	28.9 ± 13.6	28.4 ± 11.9	0.108
Number of stents	1.23 ± 0.48	1.19 ± 0.42	0.089	1.22 ± 0.47	1.15 ± 0.38	<0.001	1.20 ± 0.44	1.14 ± 0.38	<0.001

Values are means ± standard deviation or median (interquartile range) or numbers and percentages. The *p* values for continuous data obtained from the unpaired *t*-test. The *p* values for categorical data from chi-square or Fisher’s exact test. HFrEF, heart failure with reduced ejection fraction; HFmrEF, heart failure with mildly reduced ejection fraction; HFpEF, heart failure with preserved ejection fraction; NSTEMI, non-ST-segment elevation myocardial infarction; STEMI, ST-segment elevation myocardial infarction; LVEF, left ventricular ejection fraction; SBP, systolic blood pressure; DBP, diastolic blood pressure; CPR, cardiopulmonary resuscitation; SDT, symptom-to-door time; DBT, door-to-balloon time; PCI, percutaneous coronary intervention; CABG, coronary artery bypass graft; CK-MB, creatine kinase myocardial band; HDL, high-density lipoprotein; LDL, low-density lipoprotein; RASI, renin-angiotensin system inhibitor; LAD, left anterior descending artery; LCx, left circumflex artery; RCA, right coronary artery; TIMI, thrombolysis in myocardial infarction; ACC/AHA, American College of Cardiology/American Heart Association; IVUS, intravascular ultrasound; OCT, optical coherence tomography; FFR, fractional flow reserve. ^a^ Newer-generation drug-eluting stents included in the study were the zotarolimus-eluting stent (Resolute Integrity stent; Medtronic, Inc. Minneapolis, Minnesota.), everolimus-eluting stent (Xience Prime stent; Abbott Vascular or Promus Element stent; Boston Scientific Marlborough, Massachusetts.), and biolimus-eluting stent (BioMatrix Flex stent; Biosensors International or Nobori stent; Terumo Corporation, Tokyo, Japan.).

**Table 2:** In-hospital mortality between the STEMI and NSTEMI groups across all three LVEF subgroups.

	**HFrEF (LVEF ≤ 40%), *n* = 1250**						
**Outcomes**	**Group A** **NSTEMI** **(*n* = 496)**	**Group B** **STEMI ** **(*n* = 754)**	**Log-rank**	**Unadjusted**		**Adjusted ^a^**	
				**HR (95% CI)**	***p* value**	**HR (95% CI)**	***p* value**
All-cause death	31 (6.3)	46 (6.1)	0.897	1.030 (0.653–1.625)	0.897	1.011 (0.610–1.617)	0.968
Cardiac death	25 (5.1)	36 (4.8)	0.818	1.062 (0.637–1.768)	0.818	1.183 (0.667–2.096)	0.566
Non-cardiac death	6 (1.2)	10 (1.3)	0.868	0.918 (0.334–2.526)	0.869	0.610 (0.199–1.867)	0.386
	**HFmrEF (LVEF 41–49%), *n* = 2383**						
**Outcomes**	**Group C** **NSTEMI ** **(*n* = 805)**	**Group D** **STEMI ** **(*n* = 1578)**	**Log-rank**	**Unadjusted**		**Adjusted ^a^**	
				**HR (95% CI)**	***p* value**	**HR (95% CI)**	***p* value**
All-cause death	8 (1.0)	17 (1.1)	0.849	0.922 (0.398–2.136)	0.849	0.726 (0.299–1.763)	0.480
Cardiac death	4 (0.5)	12 (0.8)	0.457	0.653 (0.211–2.024)	0.460	0.879 (0.273–2.833)	0.829
Non-cardiac death	4 (0.5)	5 (0.3)	0.500	1.567 (0.421–5.835)	0.503	2.569 (0.628–10.51)	0.189
	**HFpEF (≥50%), *n* = 6221**						
**Outcomes**	**Group E** **NSTEMI ** **(*n* = 3293)**	**Group F** **STEMI ** **(*n* = 2928)**	**Log-rank**	**Unadjusted**		**Adjusted ^a^**	
				**HR (95% CI)**	***p* value**	**HR (95% CI)**	***p* value**
All-cause death	33 (1.0)	115 (3.9)	<0.001	0.251 (0.171–0.370)	<0.001	0.380 (0.244–0.591)	0.001
Cardiac death	25 (0.8)	105 (3.5)	<0.001	0.209 (0.135–0.323)	<0.001	0.309 (0.187–0.511)	<0.001
Non-cardiac death	8 (0.2)	10 (0.4)	0.434	0.691 (0.273–1.751)	0.436	0.803 (0.279–2.314)	0.685
	**Total (*n* = 9854)**						
**Outcomes**	**NSTEMI ** **(*n* = 4594)**	**STEMI ** **(*n* = 5260)**	**Log-rank**	**Unadjusted**		**Adjusted ^a^**	
				**HR (95% CI)**	***p* value**	**HR (95% CI)**	***p* value**
All-cause death	72 (1.6)	178 (3.4)	<0.001	0.458 (0.349–0.603)	<0.001	0.671 (0.497–0.907)	0.009
Cardiac death	54 (1.2)	153 (2.9)	<0.001	0.400 (0.294–0.546)	<0.001	0.601 (0.426–0.847)	0.004
Non-cardiac death	18 (0.4)	25 (0.5)	0.497	0.811 (0.442–1.486)	0.497	0.923 (0.481–1.768)	0.808

HFrEF, heart failure with reduced ejection fraction; HFmrEF, heart failure with mildly reduced ejection fraction; HFpEF, heart failure with preserved ejection fraction; NSTEMI, non-ST-segment elevation myocardial infarction; STEMI, ST-segment elevation myocardial infarction; HR, hazard ratio; CI, confidence interval; SBP, systolic blood pressure; DBP, diastolic blood pressure; BMI, body mass index; CPR, cardiopulmonary resuscitation; SDT, symptom-to-door time; DBT, door-to-balloon time; DM, diabetes mellitus; PCI, percutaneous coronary intervention; CABG, coronary artery bypass graft; CK-MB, peak creatine kinase myocardial band; HDL, high-density lipoprotein. ^a^ Adjusted by male sex, age, SBP, DBP, heart rate, BMI, Killip class II/III, cardiogenic shock, CPR on admission, SDT, DBT, hypertension, DM, dyslipidemia, previous MI, previous PCI, previous CABG, previous stroke, current smoker, peak CK-MB, peak troponin-I, hemoglobin, blood glucose, serum creatinine, triglyceride, HDL-cholesterol, and aspirin (Table S1).

**Table 3:** Comparison of 3-year outcomes between the STEMI and NSTEMI groups based on LVEF groups in the total study population.

	**HFrEF (LVEF ≤ 40%), *n* = 1250**								
**Outcomes**	**Group A** **NSTEMI** **(*n* = 496)**	**Group B** **STEMI** **(*n* = 754)**	**Log-Rank**	**Unadjusted**		**Multivariable-Adjusted ^a^**		**Propensity Score-Adjusted**	
				**HR (95% CI)**	***p* Value**	**HR (95% CI)**	***p* Value**	**HR (95% CI)**	***p* Value**
POCO	173 (34.9)	189 (25.1)	<0.001	1.465 (1.192–1.801)	<0.001	1.447 (1.161–1.757)	0.001	1.438 (1.156–1.709)	0.002
All-cause death	130 (26.2)	122 (16.2)	<0.001	1.690 (1.320–2.164)	<0.001	1.760 (1.349–2.297)	<0.001	1.740 (1.332–2.273)	<0.001
Cardiac death	90 (18.1)	94 (12.7)	0.005	1.514 (1.134–2.021)	0.005	1.674 (1.225–2.288)	0.001	1.660 (1.212–2.274)	0.002
Non-cardiac death	40 (8.1)	28 (3.5)	0.001	2.286 (1.410–3.705)	0.001	2.046 (1.231–3.400)	0.006	2.021 (1.216–3.359)	0.007
Recurrent MI	30 (7.0)	20 (3.0)	0.001	2.429 (1.379–4.277)	0.001	2.407 (1.331–4.075)	0.004	2.396 (1.297–4.038)	0.005
Any repeat revascularization	53 (12.8)	58 (8.7)	0.031	1.503 (1.036–2.182)	0.032	1.427 (0.962–2.116)	0.077	1.436 (0.967–2.135)	0.073
Hospitalization for HF	52 (11.9)	74 (10.9)	0.519	1.124 (0.788–1.602)	0.520	1.056 (0.729–1.531)	0.772	1.087 (0.748–1.579)	0.663
Stroke	20 (4.9)	20 (3.0)	0.113	1.642 (0.883–3.052)	0.117	1.638 (0.872–3.012)	0.123	1.612 (0.834–2.993)	0.155
	**HFmrEF (LVEF 41–49%), *n* = 2383**								
**Outcomes**	**Group C** **NSTEMI** **(*n* = 805)**	**Group D** **STEMI** **(*n* = 1578)**	**Log-Rank**	**Unadjusted**		**Multivariable-Adjusted ^a^**		**Propensity Score-Adjusted**	
				**HR (95% CI)**	***p* Value**	**HR (95% CI)**	***p* Value**	**HR (95% CI)**	***p* Value**
POCO	147 (18.3)	223 (14.1)	0.009	1.318 (1.070–1.623)	0.009	1.278 (1.026–1.593)	0.029	1.296 (1.041–1.621)	0.021
All-cause death	80 (9.9)	93 (5.9)	<0.001	1.714 (1.271–2.311)	<0.001	1.699 (1.237–2.333)	0.001	1.741 (1.265–2.395)	0.001
Cardiac death	42 (5.2)	51 (3.3)	0.017	1.639 (1.089–2.466)	0.018	1.643 (1.095–2.551)	0.015	1.676 (1.100–2.605)	0.011
Non-cardiac death	38 (4.7)	42 (2.9)	0.007	1.805 (1.164–2.800)	0.008	1.769 (1.118–2.699)	0.012	1.822 (1.146–2.897)	0.010
Recurrent MI	27 (3.5)	45 (3.0)	0.463	1.195 (0.742–1.926)	0.464	1.050 (0.640–1.724)	0.845	1.069 (0.652–1.758)	0.793
Any repeat revascularization	68 (8.9)	127 (8.3)	0.663	1.068 (0.795–1.433)	0.663	1.043 (0.766–1.420)	0.790	1.054 (0.773–1.436)	0.741
Hospitalization for HF	32 (4.1)	46 (3.0)	0.150	1.391 (0.886–2.184)	0.152	1.542 (0.962–2.471)	0.072	1.554 (0.969–2.493)	0.067
Stroke	18 (2.3)	28 (1.8)	0.400	1.288 (0.713–2.329)	0.402	1.246 (0.668–2.275)	0.489	1.239 (0.658–2.185)	0.510
	**HFpEF (≥50%), *n* = 6221**								
**Outcomes**	**Group E** **NSTEMI** **(*n* = 3293)**	**Group F** **STEMI** **(*n* = 2928)**	**Log-Rank**	**Unadjusted**		**Multivariable-Adjusted ^a^**		**Propensity Score-Adjusted**	
				**HR (95% CI)**	***p* Value**	**HR (95% CI)**	***p* Value**	**HR (95% CI)**	***p* Value**
POCO	456 (13.8)	484 (16.5)	0.002	0.813 (0.716–0.924)	0.002	0.901 (0.784–1.035)	0.140	0.909 (0.791–1.045)	0.181
All-cause death	174 (5.3)	238 (8.1)	<0.001	0.635 (0.522–0.772)	<0.001	0.783 (0.631–0.971)	0.026	0.776 (0.626–0.963)	0.021
Cardiac death	95 (2.9)	178 (6.1)	<0.001	0.465 (0.362–0.596)	<0.001	0.607 (0.461–0.798)	<0.001	0.603 (0.459–0.783)	<0.001
Non-cardiac death	79 (2.4)	60 (2.0)	0.447	1.139 (0.814–1.593)	0.448	1.262 (0.874–1.822)	0.214	1.238 (0.858–1.787)	0.253
Recurrent MI	90 (2.8)	71 (2.6)	0.561	1.097 (0.803–1.497)	0.561	1.081 (0.776–1.476)	0.645	1.077 (0.773–1.461)	0.662
Any repeat revascularization	272 (8.5)	239 (8.7)	0.828	0.981 (0.824–1.167)	0.828	0.977 (0.812–1.156)	0.807	0.980 (0.814–1.179)	0.828
Hospitalization for HF	58 (1.8)	44 (1.6)	0.510	1.141 (0.771–1.688)	0.510	1.091 (0.722–1.649)	0.679	1.076 (0.712–1.626)	0.729
Stroke	63 (2.0)	45 (1.6)	0.323	1.212 (0.827–1.777)	0.324	1.139 (0.757–1.714)	0.533	1.129 (0.738–1.701)	0.589
	**Total (*n* = 9854)**								
**Outcomes**	**NSTEMI** **(*n* = 4594)**	**STEMI** **(*n* = 5260)**	**Log-Rank**	**Unadjusted**		**Multivariable-Adjusted ^a^**		**Propensity Score-Adjusted**	
				**HR (95% CI)**	***p* Value**	**HR (95% CI)**	***p* Value**	**HR (95% CI)**	***p* Value**
POCO	776 (16.9)	896 (17.0)	0.681	0.980 (0.890–1.079)	0.681	0.943 (0.851–1.045)	0.264	0.927 (0.836–1.027)	0.147
All-cause death	384 (8.4)	453 (8.6)	0.566	0.961 (0.839–1.101)	0.567	0.879 (0.760–1.018)	0.085	0.864 (0.746–1.001)	0.051
Cardiac death	227 (5.0)	323 (6.1)	0.009	0.797 (0.672–0.944)	0.009	0.989 (0.824–1.188)	0.905	0.993 (0.826–1.193)	0.938
Non-cardiac death	157 (3.4)	130 (2.5)	0.008	1.369 (1.085–1.728)	0.008	1.470 (1.149–1.882)	0.002	1.487 (1.161–1.904)	0.002
Recurrent MI	147 (3.4)	136 (2.7)	0.085	1.227 (0.972–1.549)	0.086	1.217 (0.953–1.534)	0.116	1.209 (0.941–1.519)	0.214
Any repeat revascularization	393 (9.0)	424 (8.6)	0.485	1.050 (0.915–1.205)	0.485	1.037 (0.897–1.199)	0.621	1.043 (0.902–1.206)	0.570
Hospitalization for HF	142 (3.2)	164 (3.3)	0.854	0.979 (0.782–1.226)	0.854	0.998 (0.790–1.261)	0.986	0.975 (0.771–1.234)	0.833
Stroke	101 (2.3)	93 (1.9)	0.143	1.234 (0.931–1.635)	0.143	1.195 (0.887–1.611)	0.242	1.163 (0.845–1.587)	0.312

HFrEF, heart failure with reduced ejection fraction; HFmrEF, heart failure with mildly reduced ejection fraction; HFpEF, heart failure with preserved ejection fraction; NSTEMI, non-ST-segment elevation myocardial infarction; STEMI, ST-segment elevation myocardial infarction; HR, hazard ratio; CI, confidence interval; POCO, patient-oriented composite outcome; SBP, systolic blood pressure; DBP, diastolic blood pressure; BMI, body mass index; CPR, cardiopulmonary resuscitation; SDT, symptom-to-door time; DBT, door-to-balloon time; DM, diabetes mellitus; PCI, percutaneous coronary intervention; CABG, coronary artery bypass graft; CK-MB, peak creatine kinase myocardial band; HDL, high-density lipoprotein. ^a^ Adjusted by male sex, age, SBP, DBP, heart rate, BMI, Killip class II/III, cardiogenic shock, CPR on admission, SDT, DBT, hypertension, DM, dyslipidemia, previous MI, previous PCI, previous CABG, previous stroke, current smoker, peak CK-MB, peak troponin-I, hemoglobin, blood glucose, serum creatinine, triglyceride, HDL-cholesterol, and aspirin (Table S1).

## Data Availability

Data are contained within the article or Supplementary Materials.

## Supplementary Materials

The following supporting information can be downloaded at: https://www.mdpi.com/article/10.3390/jcm13226744/s1, Figure S1: Flowchart after exclusion of in-hospital mortality; Figure S2: Kaplan-Meier analysis of POCO (A), all-cause death (B), cardiac death (C), and non-cardiac death (D) during a 3-year follow-up period after excluding in-hospital mortality; Figure S3: Trends in the use of beta-blockers, RASI, and statin users during the 3-year follow-up period after discharge; Figure S4: Flowchart based on male and female groups; Figure S5: Flowchart based on male and female groups after exclusion of in-hospital mortality; Table S1: Results of collinearity testing for POCO between the NSTEMI and STEMI groups; Table S2: Baseline characteristics of patients with NSTEMI; Table S3: Baseline characteristics of patients with STEMI; Table S4: Baseline characteristics of the total study population before and after propensity-score matched analysis; Table S5: Comparison of 3-year mortality between the STEMI and NSTEMI groups based on LVEF groups after excluding in-hospital mortality; Table S6: Comparison of 3-year outcomes among three LVEF subgroups in NSTEMI and STEMI; Table S7: Comparison of 3-year outcomes among three LVEF subgroups in NSTEMI and STEMI after excluding in-hospital mortality; Table S8: Independent predictors for POCO and all-cause death in the total study population; Table S9: Independent predictors for POCO and all-cause death among patients excluding in-hospital mortality; Table S10: In-hospital mortality between the STEMI and NSTEMI groups across three LVEF subgroups in male patients; Table S11: In-hospital mortality between the STEMI and NSTEMI groups across three LVEF subgroups in female patients; Table S12: Comparison of 3-year mortality between the STEMI and NSTEMI groups based on LVEF groups in male patients; Table S13: Comparison of 3-year mortality between the STEMI and NSTEMI groups based on LVEF groups in female patients; Table S14: Comparison of 3-year mortality between the STEMI and NSTEMI groups based on LVEF subgroups after excluding in-hospital mortality in male patients; Table S15: Comparison of 3-year mortality between the STEMI and NSTEMI groups based on LVEF subgroups after excluding in-hospital mortality in female patients.

## References

[1] Thygesen K., Alpert J.S., Jaffe A.S., Chaitman B.R., Bax J.J., Morrow D.A., White H.D. (2018). Fourth Universal Definition of Myocardial Infarction. J. Am. Coll. Cardol..

[2] Goldberg R.J., Currie K., White K., Brieger D., Steg P.G., Goodman S.G., Dabbous O., Fox K.A., Gore J.M. (2004). Six-month outcomes in a multinational registry of patients hospitalized with an acute coronary syndrome (the Global Registry of Acute Coronary Events [GRACE]). Am. J. Cardiol..

[3] Chan M.Y., Sun J.L., Newby L.K., Shaw L.K., Lin M., Peterson E.D., Califf R.M., Kong D.F., Roe M.T. (2009). Long-term mortality of patients undergoing cardiac catheterization for ST-elevation and non-ST-elevation myocardial infarction. Circulation.

[4] Li Z., Huang S., Yang R., Li J., Chen G. (2021). Long-term follow-up of diabetic patients with non-ST-segment elevation myocardial infarction. Am. J. Transl. Res..

[5] Krishnamurthy S.N., Pocock S., Kaul P., Owen R., Goodman S.G., Granger C.B., Nicolau J.C., Simon T., Westermann D., Yasuda S. (2023). Comparing the long-term outcomes in chronic coronary syndrome patients with prior ST-segment and non-ST-segment elevation myocardial infarction: Findings from the TIGRIS registry. BMJ Open.

[6] Pilgrim T., Vranckx P., Valgimigli M., Stefanini G.G., Piccolo R., Rat J., Rothenbühler M., Stortecky S., Räber L., Blöchlinger S. (2016). Risk and timing of recurrent ischemic events among patients with stable ischemic heart disease, non-ST-segment elevation acute coronary syndrome, and ST-segment elevation myocardial infarction. Am. Heart J..

[7] Fox C.S., Muntner P., Chen A.Y., Alexander K.P., Roe M.T., Cannon C.P., Saucedo J.F., Kontos M.C., Wiviott S.D. (2010). Use of evidence-based therapies in short-term outcomes of ST-segment elevation myocardial infarction and non-ST-segment elevation myocardial infarction in patients with chronic kidney disease: A report from the National Cardiovascular Data Acute Coronary Treatment and Intervention Outcomes Network registry. Circulation.

[8] Members W.C. (2022). 2022 AHA/ACC/HFSA Guideline for the Management of Heart Failure. J. Card. Fail..

[9] Jenča D., Melenovský V., Stehlik J., Staněk V., Kettner J., Kautzner J., Adámková V., Wohlfahrt P. (2021). Heart failure after myocardial infarction: Incidence and predictors. ESC Heart Fail..

[10] Minicucci M.F., Azevedo P.S., Polegato B.F., Paiva S.A., Zornoff L.A. (2011). Heart failure after myocardial infarction: Clinical implications and treatment. Clin. Cardiol..

[11] Wang T.J., Evans J.C., Benjamin E.J., Levy D., LeRoy E.C., Vasan R.S. (2003). Natural history of asymptomatic left ventricular systolic dysfunction in the community. Circulation.

[12] Ponikowski P., Voors A.A., Anker S.D., Bueno H., Cleland J.G.F., Coats A.J.S., Falk V., González-Juanatey J.R., Harjola V.P., Jankowska E.A. (2016). 2016 ESC Guidelines for the diagnosis and treatment of acute and chronic heart failure: The Task Force for the diagnosis and treatment of acute and chronic heart failure of the European Society of Cardiology (ESC)Developed with the special contribution of the Heart Failure Association (HFA) of the ESC. Eur. Heart J..

[13] Kim Y.H., Her A.Y., Jeong M.H., Kim B.K., Hong S.J., Kim J.S., Ko Y.G., Choi D., Hong M.K., Jang Y. (2020). Impact of stent generation on 2-year clinical outcomes in ST-segment elevation myocardial infarction patients with multivessel disease who underwent culprit-only or multivessel percutaneous coronary intervention. Catheter. Cardiovasc. Interv..

[14] Kim J.H., Chae S.C., Oh D.J., Kim H.S., Kim Y.J., Ahn Y., Cho M.C., Kim C.J., Yoon J.H., Park H.Y. (2016). Multicenter Cohort Study of Acute Myocardial Infarction in Korea—Interim Analysis of the Korea Acute Myocardial Infarction Registry-National Institutes of Health Registry. Circ. J..

[15] Kim H., Lee K.Y., Choo E.H., Hwang B.H., Kim J.J., Kim C.J., Chang K., Hong Y.J., Kim J.H., Ahn Y. (2024). KAMIR-NIH Investigators. Long-Term Risk of Cardiovascular Death in Patients With Mildly Reduced Ejection Fraction After Acute Myocardial Infarction: A Multicenter, Prospective Registry Study. J. Am. Heart Assoc..

[16] Grech E.D. (2003). ABC of interventional cardiology: Percutaneous coronary intervention. II: The procedure. BMJ.

[17] Collet J.P., Thiele H., Barbato E., Barthélémy O., Bauersachs J., Bhatt D.L., Dendale P., Dorobantu M., Edvardsen T., Folliguet T. (2021). 2020 ESC Guidelines for the management of acute coronary syndromes in patients presenting without persistent ST-segment elevation. Eur. Heart J..

[18] Ibanez B., James S., Agewall S., Antunes M.J., Bucciarelli-Ducci C., Bueno H., Caforio A.L.P., Crea F., Goudevenos J.A., Halvorsen S. (2018). 2017 ESC Guidelines for the management of acute myocardial infarction in patients presenting with ST-segment elevation: The Task Force for the management of acute myocardial infarction in patients presenting with ST-segment elevation of the European Society of Cardiology (ESC). Eur. Heart J..

[19] Lee J.M., Rhee T.M., Hahn J.Y., Kim H.K., Park J., Hwang D., Choi K.H., Kim J., Park T.K., Yang J.H. (2018). Multivessel Percutaneous Coronary Intervention in Patients With ST-Segment Elevation Myocardial Infarction With Cardiogenic Shock. J. Am. Coll. Cardiol..

[20] Cutlip D.E., Windecker S., Mehran R., Boam A., Cohen D.J., van Es G.A., Steg P.G., Morel M.A., Mauri L., Vranckx P. (2007). Clinical end points in coronary stent trials: A case for standardized definitions. Circulation.

[21] Vatcheva K.P., Lee M., McCormick J.B., Rahbar M.H. (2016). Multicollinearity in Regression Analyses Conducted in Epidemiologic Studies. Epidemiology.

[22] Marcoulides K.M., Raykov T. (2019). Evaluation of Variance Inflation Factors in Regression Models Using Latent Variable Modeling Methods. Educ. Psychol. Meas..

[23] Cleland J.G., Torabi A., Khan N.K. (2005). Epidemiology and management of heart failure and left ventricular systolic dysfunction in the aftermath of a myocardial infarction. Heart.

[24] Polonski L., Gasior M., Gierlotka M., Osadnik T., Kalarus Z., Trusz-Gluza M., Zembala M., Wilczek K., Lekston A., Zdrojewski T. (2011). A comparison of ST elevation versus non-ST elevation myocardial infarction outcomes in a large registry database: Are non-ST myocardial infarctions associated with worse long-term prognoses?. Int. J. Cardiol..

[25] Sawayama Y., Takashima N., Harada A., Yano Y., Yamamoto T., Higo Y., Shioyama W., Fujii T., Tanaka-Mizuno S., Kita Y. (2023). Incidence and In-Hospital Mortality of Acute Myocardial Infarction: A Report from a Population-Based Registry in Japan. J. Atheroscler. Thromb..

[26] Harjola V.P., Lassus J., Sionis A., Køber L., Tarvasmäki T., Spinar J., Parissis J., Banaszewski M., Silva-Cardoso J., Carubelli V. (2015). Clinical picture and risk prediction of short-term mortality in cardiogenic shock. Eur. J. Heart Fail..

[27] Djohan A.H., Evangelista L.K.M., Chan K.H., Lin W., Adinath A.A., Kua J.L., Sim H.W., Chan M.Y., Ng G., Cherian R. (2024). Clinical predictors and outcomes of ST-elevation myocardial infarction related cardiogenic shock in the Asian population. Int. J. Cardiol. Heart Vasc..

[28] Furtado R.H.M., Juliasz M.G., Chiu F.Y.J., Bastos L.B.C., Dalcoquio T.F., Lima F.G., Rosa R., Caporrino C.A., Bertolin A., Genestreti P.R.R. (2023). Long-term mortality after acute coronary syndromes among patients with normal, mildly reduced, or reduced ejection fraction. ESC Heart Fail..

[29] Alkhalil M., Kearney A., MacElhatton D., Fergie R., Dixon L. (2020). The prognostic role of mid-range ejection fraction in ST-segment elevation myocardial infarction. Int. J. Cardiol..

[30] Karabağ Y., Çınar T., Çağdaş M., Rencüzoğulları İ., Tanık V.O. (2019). In-hospital and long-term prognoses of patients with a mid-range ejection fraction after an ST-segment myocardial infarction. Acta Cardiol..

[31] Paradossi U., De Caterina A.R., Trimarchi G., Pizzino F., Bastiani L., Dossi F., Raccis M., Bianchi G., Palmieri C., de Gregorio C. (2024). The enigma of the ‘smoker’s paradox’: Results from a single-center registry of patients with STEMI undergoing primary percutaneous coronary intervention. Cardiovasc. Revascularization Med..

[32] Rodríguez-Padial L., Fernández-Pérez C., Bernal J.L., Anguita M., Sambola A., Fernández-Ortiz A., Elola F.J. (2021). Differences in in-hospital mortality after STEMI versus NSTEMI by sex. Eleven-year trend in the Spanish National Health Service. Rev. Española Cardiol..

[33] Pancholy S.B., Shantha G.P., Patel T., Cheskin L.J. (2014). Sex differences in short-term and long-term all-cause mortality among patients with ST-segment elevation myocardial infarction treated by primary percutaneous intervention: A meta-analysis. JAMA Intern. Med..

[34] Heidenreich P.A., Bozkurt B., Aguilar D., Allen L.A., Byun J.J., Colvin M.M., Deswal A., Drazner M.H., Dunlay S.M., Evers L.R. (2022). 2022 AHA/ACC/HFSA Guideline for the Management of Heart Failure: A Report of the American College of Cardiology/American Heart Association Joint Committee on Clinical Practice Guidelines. Circulation.

