# Supplementary material for: Comparison of Outcomes Between ST-Segment Elevation and Non-ST-Segment Elevation Myocardial Infarctions Based on Left Ventricular Ejection Fraction
Source: J Clin Med. 2024 Nov 9;13(22):6744. doi: 10.3390/jcm13226744 (PMC11595183; doi:10.3390/jcm13226744)
Supplement: Supplementary file 1 [file jcm-13-06744-s001.zip › jcm-3282418-supplementary.pdf]

Comparison of Outcomes Between ST-Segment Elevation and Non-ST-Segment Elevation Myocardial Infarctions Based on Left Ventricular Ejection Fraction

Running title: NSEMI vs. STEMI based on LVEF

Yong Hoon Kim[1,*,†], Ae-Young Her[1, †], Seung-Woon Rha[2,*], Cheol Ung Choi[2], Byoung Geol Choi[3], Soohyung Park[2], Su Jin Hyun[2], Jung Rae Cho[4], Min-Woong Kim[5], Ji Young Park[6], and Myung Ho Jeong[7]

Supplementary Online Contents

Table S1. Results of collinearity testing for POCO between the NSTEMI and STEMI groups

Table S2. Baseline characteristics of patients with NSTEMI

Table S3. Baseline characteristics of patients with STEMI

Table S4. Baseline characteristics of the total study population before and after propensity-score matched analysis.

Table S5. Comparison of 3-year mortality between the STEMI and NSTEMI groups based on LVEF groups after excluding in-hospital mortality

Table S6. Comparison of 3-year outcomes among three LVEF subgroups in NSTEMI and STEMI

Table S7. Comparison of 3-year outcomes among three LVEF subgroups in NSTEMI and STEMI after excluding in-hospital mortality

Table S8. Independent predictors for POCO and all-cause death in the total study population.

Table S9. Independent predictors for POCO and all-cause death among patients excluding in-hospital mortality.

Table S10. In-hospital mortality between the STEMI and NSTEMI groups across three LVEF subgroups in male patients.

Table S11. In-hospital mortality between the STEMI and NSTEMI groups across three LVEF subgroups in female patients.

Table S12. Comparison of 3-year mortality between the STEMI and NSTEMI groups based on LVEF groups in male patients

Table S13. Comparison of 3-year mortality between the STEMI and NSTEMI groups based on LVEF groups in female patients

Table S14. Comparison of 3-year mortality between the STEMI and NSTEMI groups based on LVEF subgroups after excluding in-hospital mortality in male patients

Table S15. Comparison of 3-year mortality between the STEMI and NSTEMI groups based on LVEF subgroups after excluding in-hospital mortality in female patients.

Figure S1. Flowchart after exclusion of in-hospital mortality

Figure S2. Kaplan-Meier analysis of POCO (A), all-cause death (B), cardiac death (C), and non-cardiac death (D) during a 3-year follow-up period after excluding in-hospital mortality.

Figure S3. Trends in the use of beta-blockers, RASI, and statin users during the 3-year follow-up period after discharge.

Figure S4. Flowchart based on male and female groups

Figure S5. Flowchart based on male and female groups after exclusion of in-hospital mortality.

Table S1. Results of collinearity testing for POCO between the NSTEMI and STEMI groups

	Variance Inflation Factors	Tolerance	Condition Index
Male	1.548	0.646	1.000
Age	1.874	0.534	3.661
Systolic blood pressure	3.144	0.318	3.899
Diastolic blood pressure	3.159	0.317	4.017
Heart rate	1.177	0.850	4.345
Body mass index	1.242	0.805	4.508
Killip class II/III	1.104	0.906	4.726
Cardiogenic shock	1.251	0.800	5.001
CPR on admission	1.235	0.810	5.171
SDT	1.038	0.964	5.195
DBT	1.105	0.905	5.254
Hypertension	1.213	0.825	5.348
Diabetes mellitus	1.445	0.692	5.382
Dyslipidemia	1.034	0.967	5.546
Previous MI	1.954	0.512	5.696
Previous PCI	1.968	0.508	5.731
Previous CABG	1.063	0.941	5.843
Previous stroke	1.045	0.957	6.116
Current smoker	1.392	0.718	6.348
Peak CK-MB	1.215	0.823	6.557
Peak troponin-I	1.180	0.848	6.908
Hemoglobin	1.937	0.516	7.160
Blood glucose	1.391	0.719	7.428
Serum creatinine	1.177	0.850	7.765
Total cholesterol	6.579	0.152	7.770
Triglyceride	1.499	0.667	8.149
HDL-cholesterol	1.357	0.737	8.282
LDL-cholesterol	5.528	0.181	8.834
Aspirin	1.087	0.920	9.646
Clopidogrel	1.328	0.753	10.725
Prasugrel	1.362	0.734	10.836
Beta-blocker	1.171	0.854	11.392
RASI	1.166	0.858	11.876
Statin	1.172	0.853	11.978
Anticoagulant	1.023	0.978	11.998
Left main (IRA)	2.887	0.346	12.070
LAD (IRA)	3.393	0.295	12.708
LCx (IRA)	5.594	0.179	13.125
RCA (IRA)	2.756	0.363	14.758
Left main (treated vessel)	3.921	0.331	15.163
LAD (treated vessel)	3.021	0.410	15.876
LCx (treated vessel)	2.436	0.256	17.075

RCA (treated vessel)	4.160	0.240	19.873
Multivessel disease	1.598	0.626	21.151
Transradial approach	1.141	0.876	22.701
Pre-PCI TIMI flow grade 0/1	1.253	0.798	25.183
ACC/AHA type B2/C lesion	1.086	0.921	27.043
Glycoprotein IIb/IIIa inhibitor	1.114	0.898	29.750
IVUS/OCT	1.098	0.911	32.270
FFR	1.021	0.980	44.471
EES	1.066	0.938	49.450
Other stent	1.025	0.976	52.753
Stent diameter	1.228	0.814	60.059
Stent length	2.512	0.398	66.455
Number of stent	2.411	0.415	124.728

POCO, patient-oriented composite outcome; NSTEMI, non-ST-segment elevation myocardial infarction; STEMI, ST-segment elevation myocardial infarction; CPR, cardiopulmonary resuscitation; SDT, symptom-to-door time; DBT, door-to-balloon time; PCI, percutaneous coronary intervention; CABG, coronary artery bypass graft; CK-MB, creatine kinase myocardial band; HDL, high-density lipoprotein; LDL, low-density lipoprotein; RASI, renin-angiotensin system inhibitor; IRA, infarct-related artery; LAD, left anterior descending coronary artery; LCx, left circumflex coronary artery; RCA, right coronary artery; TIMI, thrombolysis in myocardial infarction; ACC/AHA, American College of Cardiology/American Heart Association; IVUS, intravascular ultrasound; OCT, optical coherence tomography; FFR, fractional flow reserve; EES, everolimus-eluting stent.

Table S2. Baseline characteristics of patients with NSTEMI

Variables	HFrEF (*n* = 496) Group A	HFmrEF (*n* = 805) Group C	HFpEF (*n* = 3293) Group E	*p* value			
				Group A vs. C	Group A vs. E	Group C vs. E	Group A vs. C vs. E
Male, *n* (%)	326 (65.7)	557 (69.2)	2452 (74.5)	0.200	<0.001	0.003	<0.001
Age, years	69.1 ± 11.1	66.5 ± 11.7	63.0 ± 12.0	<0.001	<0.001	<0.001	<0.001
SBP, mmHg	131.9 ± 27.6	131.8 ± 25.3	137.1 ± 25.9	0.964	<0.001	<0.001	<0.001
DBP, mmHg	79.9 ± 16.7	79.5 ± 15.7	82.0 ± 15.1	0.709	0.008	<0.001	<0.001
Heart rate, beats/min	92.4 ± 20.0	80.5 ± 17.6	81.9 ± 15.1	<0.001	<0.001	<0.001	<0.001
Body mass index, Kg/m^2	23.4 ± 3.8	23.7 ± 3.2	24.3 ± 3.3	0.128	<0.001	<0.001	<0.001
LVEF, %	32.7 ± 6.3	45.8 ± 2.5	59.5 ± 6.2	<0.001	<0.001	<0.001	<0.001
Killip class II/III, *n* (%)	229 (46.2)	158 (19.6)	323 (9.8)	<0.001	<0.001	<0.001	<0.001
Cardiogenic shock, *n* (%)	18 (3.6)	14 (1.7)	52 (1.6)	0.042	0.004	0.756	0.006
CPR on admission, *n* (%)	59 (11.9)	29 (3.6)	54 (1.6)	0.031	<0.001	0.001	<0.001
SDT, hours	11.1 (3.3–48.0)	7.6 (2.7–25.8)	6.1 (2.2–24.0)	0.031	0.058	0.596	0.180
DBT, hours	15.5 (3.6–39.5)	13.0 (3.4–24.2)	13.7 (4.0–24.4)	<0.001	<0.001	0.172	<0.001
Risk factors							
Hypertension, *n* (%)	305 (61.5)	405 (50.3)	1730 (52.5)	<0.001	<0.001	0.271	<0.001
Diabetes mellitus, *n* (%)	238 (48.0)	262 (32.5)	917 (27.8)	<0.001	<0.001	0.009	<0.001
Dyslipidemia, *n* (%)	43 (8.7)	67 (8.3)	447 (13.6)	0.838	0.002	<0.001	<0.001
Previous MI, *n* (%)	69 (13.9)	69 (8.6)	172 (5.2)	0.003	<0.001	0.001	<0.001
Previous PCI, *n* (%)	81 (16.3)	85 (10.6)	285 (8.7)	0.003	<0.001	0.099	<0.001
Previous CABG, *n* (%)	11 (2.2)	8 (1.0)	16 (0.5)	0.095	<0.001	0.117	<0.001
Previous stroke, *n* (%)	59 (11.9)	56 (7.0)	168 (5.1)	0.003	<0.001	0.046	<0.001
Current smokers, *n* (%)	135 (27.2)	271 (33.7)	1248 (37.9)	0.016	<0.001	0.028	<0.001
Laboratory results							
Peak CK-MB, mg/dL	21.4 (6.4–68.3)	35.0 (8.4–125.8)	18.6 (5.5–70.5)	<0.001	0.274	<0.001	<0.001
Peak troponin-I, ng/mL	8.9 (2.5–31.5)	10.8 (2.5–31.8)	6.0 (1.3–21.5)	0.582	<0.001	<0.001	<0.001
Hemoglobin, mg/dL	12.4 ± 2.4	13.4 ± 1.9	13.9 ± 1.9	<0.001	<0.001	<0.001	<0.001
Blood glucose, mg/dL	197.7 ± 86.3	164.3 ± 84.2	152.3 ± 69.7	<0.001	<0.001	<0.001	<0.001
Serum creatinine, mg/dL	1.75 ± 0.88	1.17 ± 0.88	1.05 ± 0.75	<0.001	<0.001	0.021	<0.001

Total cholesterol, mg/dL	169.0 ± 45.6	176.6 ± 46.7	181.5 ± 44.3	0.005	0.001	0.008	<0.001
Triglyceride, mg/dL	113.1 ± 98.6	121.3 ± 90.4	138.0 ± 79.4	0.098	<0.001	<0.001	<0.001
HDL-cholesterol, mg/dL	42.3 ± 12.8	42.5 ± 11.8	42.8 ± 11.4	0.832	0.429	0.467	0.575
LDL-cholesterol, mg/dL	103.9 ± 39.5	110.9 ± 39.5	115.2 ± 38.3	0.005	<0.001	0.009	<0.001
Discharge medications							
Aspirin, n (%)	483 (97.4)	796 (98.9)	3254 (98.8)	0.048	0.020	0.875	0.028
Clopidogrel, *n* (%)	395 (79.6)	607 (75.4)	2328 (70.7)	0.090	<0.001	0.008	<0.001
Ticagrelor, *n* (%)	71 (14.3)	135 (16.8)	641 (19.5)	0.274	0.006	0.088	0.009
Prasugrel, *n* (%)	30 (6.0)	63 (7.8)	324 (9.8)	0.268	0.006	0.081	0.009
Beta-blocker, *n* (%)	395 (79.6)	681 (84.6)	2803 (85.1)	0.024	0.002	0.700	0.007
RASI, *n* (%)	389 (78.4)	642 (79.8)	2732 (83.0)	0.574	0.016	0.035	0.011
Statin, *n* (%)	434 (87.5)	761 (94.5)	3133 (95.1)	<0.001	<0.001	0.470	<0.001
Anticoagulant, *n* (%)	36 (7.3)	21 (2.6)	30 (0.9)	<0.001	<0.001	<0.001	<0.001
Infarct-related artery							
Left main, *n* (%)	26 (5.2)	20 (2.5)	97 (2.9)	0.013	0.013	0.555	0.012
LAD, *n* (%)	257 (51.8)	413 (51.3)	1295 (39.3)	0.864	<0.001	<0.001	<0.001
LCx, *n* (%)	78 (15.7)	181 (22.5)	909 (27.6)	0.003	<0.001	0.003	<0.001
RCA, *n* (%)	135 (27.2)	191 (23.7)	992 (30.1)	0.167	0.206	<0.001	0.001
Treated vessel							
Left main, *n* (%)	36 (7.3)	38 (4.7)	146 (4.4)	0.064	0.009	0.705	0.023
LAD, *n* (%)	352 (71.0)	520 (64.6)	1786 (54.2)	0.018	<0.001	<0.001	<0.001
LCx, *n* (%)	170 (34.3)	287 (35.7)	1320 (40.1)	0.633	0.014	0.022	0.007
RCA, *n* (%)	195 (39.3)	272 (33.8)	1268 (38.5)	0.050	0.729	0.013	0.035
Multivessel disease, *n* (%)	351 (70.8)	475 (59.0)	1744 (53.0)	<0.001	<0.001	0.002	<0.001
Transradial approach, *n* (%)	208 (41.9)	378 (47.0)	1775 (53.9)	0.085	<0.001	<0.001	<0.001
Pre-PCI TIMI flow grade 0/1, *n* (%)	203 (40.9)	382 (47.5)	1211 (36.8)	0.022	0.081	<0.001	<0.001
ACC/AHA type B2/C lesion, *n* (%)	434 (87.5)	707 (87.8)	2742 (83.8)	0.862	0.018	0.001	0.001
Glycoprotein IIb/IIIa inhibitor, *n* (%)	35 (7.1)	75 (9.3)	294 (8.9)	0.182	0.199	0.731	0.330
IVUS/OCT, *n* (%)	92 (18.5)	208 (25.8)	869 (26.4)	0.002	<0.001	0.789	0.001
FFR, *n* (%)	6 (1.2)	13 (1.6)	86 (2.6)	0.640	0.060	0.123	0.056
Drug-eluting stents [a]							
ZES, *n* (%)	118 (23.8)	190 (23.6)	773 (23.5)	0.947	0.865	0.963	0.987

EES, *n* (%)	289 (58.3)	430 (53.4)	1667 (50.6)	0.096	0.002	0.157	0.004
BES, *n* (%)	68 (13.7)	152 (18.9)	700 (21.3)	0.018	<0.001	0.146	<0.001
Others, *n* (%)	21 (4.2)	33 (4.1)	153 (4.6)	0.887	0.818	0.571	0.761
Stent diameter, mm	3.06 ± 0.41	3.05 ± 0.39	3.08 ± 0.43	0.625	0.254	0.031	0.092
Stent length, mm	31.8 ± 15.4	31.1 ± 14.9	28.9 ± 13.6	0.393	<0.001	<0.001	<0.001
Number of stents	1.23 ± 0.48	1.22 ± 0.47	1.20 ± 0.44	0.693	0.111	0.158	0.117

Values are means ± standard deviation or median (interquartile range) or numbers and percentages. The *p* Values for continuous data were obtained using analysis of variance or the Jonckheere-Terpstra test for comparisons among three groups, and using the unpaired t-test for comparisons between two groups. The *p* values for categorical data from chi-square or Fisher's exact test. HFrEF, heart failure with reduced ejection fraction; HFmrEF, heart failure with mildly reduced ejection fraction; HFpEF, heart failure with preserved ejection fraction; NSTEMI, non-ST-segment elevation myocardial infarction; STEMI, ST-segment elevation myocardial infarction; LVEF, left ventricular ejection fraction; SBP, systolic blood pressure; DBP, diastolic blood pressure; CPR, cardiopulmonary resuscitation; SDT, symptom-to-door time; DBT, door-to-balloon time; PCI, percutaneous coronary intervention; CABG, coronary artery bypass graft; CK-MB, creatine kinase myocardial band; HDL, high-density lipoprotein; LDL, low-density lipoprotein; RASI, renin-angiotensin system inhibitor; LAD, left anterior descending artery; LCx, left circumflex artery; RCA, right coronary artery; TIMI, thrombolysis in myocardial infarction; ACC/AHA, American College of Cardiology/American Heart Association; IVUS, intravascular ultrasound; OCT, optical coherence tomography; FFR, fractional flow reserve; ZES, zotarolimus-eluting stent; EES, everolimus-eluting stent; BES, biolimus-eluting stent. [a] Newer-generation drug-eluting stents included in the study were the zotarolimus-eluting stent (Resolute Integrity stent; Medtronic, Inc.), everolimus-eluting stent (Xience Prime stent; Abbott Vascular or Promus Element stent; Boston Scientific), and biolimus-eluting stent (BioMatrix Flex stent; Biosensors International or Nobori stent; Terumo Corporation).

Table S3. Baseline characteristics of patients with STEMI

Variables	HFrEF (*n* = 754) Group B	HFmrEF (*n* = 1578) Group D	HFpEF (*n* = 2928) Group F	*p* value			
				Group B vs. D	Group B vs. F	Group D vs. F	Group B vs. D vs. F
Male, n (%)	567 (75.2)	1232 (78.1)	2346 (80.1)	0.126	0.004	0.105	0.009
Age, years	64.9 ± 12.8	62.3 ± 12.4	61.6 ± 12.5	<0.001	<0.001	0.062	<0.001
SBP, mmHg	124.6 ± 27.8	129.6 ± 26.8	127.8 ± 28.2	<0.001	0.006	0.035	<0.001
DBP, mmHg	78.1 ± 17.9	79.2 ± 16.5	77.8 ± 17.6	0.171	0.706	0.011	0.044
Heart rate, beats/min	86.3 ± 20.3	78.6 ± 18.1	74.2 ± 18.9	<0.001	<0.001	<0.001	<0.001
Body mass index, Kg/m^2	23.5 ± 3.3	24.0 ± 3.3	24.3 ± 3.1	0.001	<0.001	0.033	<0.001
LVEF, %	33.5 ± 6.1	45.6 ± 2.4	57.6 ± 5.8	<0.001	<0.001	<0.001	<0.001
Killip class II/III, *n* (%)	187 (24.8)	243 (15.4)	275 (9.4)	<0.001	<0.001	<0.001	<0.001
Cardiogenic shock, *n* (%)	61 (8.1)	81 (5.1)	233 (8.0)	0.007	0.881	<0.001	0.001
CPR on admission, *n* (%)	111 (14.7)	75 (4.8)	187 (6.4)	<0.001	<0.001	0.028	<0.001
SDT, hours	3.1 (1.5–9.9)	2.8 (1.2–6.5)	2.0 (1.0–4.6)	0.040	<0.001	0.002	<0.001
DBT, hours	0.9 (0.8–1.3)	1.0 (0.8–1.3)	0.9 (0.8–1.3)	0.264	0.622	0.414	0.802
Risk factors							
Hypertension, *n* (%)	349 (46.3)	707 (44.8)	1355 (46.3)	0.505	0.996	0.347	0.616
Diabetes mellitus, *n* (%)	246 (32.6)	379 (24.0)	658 (22.5)	<0.001	<0.001	0.250	<0.001
Dyslipidemia, *n* (%)	75 (9.9)	160 (10.1)	318 (10.9)	0.941	0.509	0.478	0.648
Previous MI, *n* (%)	59 (7.8)	82 (5.2)	99 (3.4)	0.016	<0.001	0.004	<0.001
Previous PCI, *n* (%)	72 (9.5)	97 (6.1)	151 (5.2)	0.004	<0.001	0.171	<0.001
Previous CABG, *n* (%)	4 (0.5)	6 (0.4)	8 (0.3)	0.736	0.282	0.580	0.533
Previous stroke, *n* (%)	51 (6,8)	63 (4.0)	105 (3.6)	0.005	<0.001	0.510	<0.001
Current smokers, *n* (%)	306 (40.6)	732 (46.4)	1342 (45.8)	0.009	0.010	0.730	0.020
Laboratory results							
Peak CK-MB, mg/dL	156.5 (26.0–300.0)	172.0 (50.4–311.5)	104.3 (29.4–224.1)	0.005	<0.001	<0.001	<0.001
Peak troponin-I, ng/mL	54.4 (16.3–168.8)	47.7 (20.6–111.1)	27.9 (11.5–71.9)	<0.001	<0.001	<0.001	<0.001
Hemoglobin, mg/dL	13.8 ± 2.0	14.3 ± 2.0	14.3 ± 1.9	<0.001	<0.001	0.336	<0.001
Blood glucose, mg/dL	199.1 ± 96.1	175.8 ± 75.8	173.6 ± 76.3	<0.001	<0.001	0.359	<0.001
Serum creatinine, mg/dL	1.12 ± 0.79	1.01 ± 0.83	1.00 ± 0.63	0.002	<0.001	0.614	<0.001

Total cholesterol, mg/dL	176.5 ± 46.7	185.5 ± 44.1	182.6 ± 43.8	<0.001	0.001	0.042	<0.001
Triglyceride, mg/dL	120.7 ± 94.9	134.9 ± 88.8	147.9 ± 82.3	0.001	<0.001	<0.001	<0.001
HDL-cholesterol, mg/dL	43.9 ± 14.6	43.5 ± 11.9	41.9 ± 11.7	0.519	0.001	<0.001	<0.001
LDL-cholesterol, mg/dL	111.5 ± 44.5	117.7 ± 38.8	115.8 ± 38.2	0.002	0.025	0.130	0.004
Discharge medications							
Aspirin, n (%)	729 (96.7)	1565 (99.2)	2862 (97.7)	<0.001	0.113	<0.001	<0.001
Clopidogrel, *n* (%)	558 (74.0)	1062 (67.4)	1944 (66.4)	0.001	<0.001	0.551	<0.001
Ticagrelor, *n* (%)	136 (18.0)	314 (19.9)	620 (21.2)	0.313	0.061	0.317	0.141
Prasugrel, *n* (%)	60 (8.0)	202 (12.8)	364 (12.4)	<0.001	<0.001	0.742	0.001
Beta-blocker, *n* (%)	595 (78.9)	1407 (89.2)	2509 (85.7)	<0.001	<0.001	0.001	<0.001
RASI, *n* (%)	566 (75.1)	1298 (82.3)	2308 (78.8)	<0.001	0.030	0.006	<0.001
Statin, *n* (%)	663 (87.9)	1489 (94.4)	2712 (92.6)	<0.001	<0.001	0.029	<0.001
Anticoagulant, *n* (%)	47 (6.2)	53 (3.4)	44 (1.5)	0.002	<0.001	<0.001	<0.001
Infarct-related artery							
Left main, *n* (%)	32 (4.2)	18 (1.1)	25 (0.9)	<0.001	<0.001	0.340	<0.001
LAD, *n* (%)	549 (72.8)	1028 (65.1)	1165 (39.8)	<0.001	<0.001	<0.001	<0.001
LCx, *n* (%)	30 (4.0)	127 (8.0)	300 (10.2)	<0.001	<0.001	0.016	<0.001
RCA, *n* (%)	143 (19.0)	405 (25.7)	1438 (49.1)	<0.001	<0.001	<0.001	<0.001
Treated vessel							
Left main, *n* (%)	39 (5.2)	32 (2.0)	44 (1.5)	<0.001	<0.001	0.225	<0.001
LAD, *n* (%)	620 (82.2)	1127 (71.4)	1455 (49.7)	<0.001	<0.001	<0.001	<0.001
LCx, *n* (%)	118 (15.6)	254 (16.1)	536 (18.3)	0.809	0.098	0.065	0.078
RCA, *n* (%)	203 (26.9)	497 (31.5)	1568 (53.6)	0.026	<0.001	<0.001	<0.001
Multivessel disease, *n* (%)	423 (56.1)	733 (46.5)	1342 (45.8)	<0.001	<0.001	0.707	<0.001
Transradial approach, *n* (%)	185 (24.5)	444 (28.1)	739 (25.2)	0.073	0.707	0.036	0.065
Pre-PCI TIMI flow grade 0/1, *n* (%)	580 (76.9)	1209 (76.6)	2107 (72.0)	0.917	0.007	0.001	<0.001
ACC/AHA type B2/C lesion, *n* (%)	683 (90.6)	1399 (88.7)	2577 (88.0)	0.174	0.054	0.529	0.140
Glycoprotein IIb/IIIa inhibitor, *n* (%)	130 (17.2)	322 (20.4)	651 (22.2)	0.073	0.003	0.160	0.009
IVUS/OCT, *n* (%)	133 (17.6)	316 (20.0)	597 (20.4)	0.178	0.101	0.786	0.238
FFR, *n* (%)	5 (0.7)	9 (0.6)	24 (0.8)	0.779	0.819	0.464	0.628
Drug-eluting stents [a]							
ZES, *n* (%)	170 (22.5)	363 (23.0)	679 (23.2)	0.833	0.734	0.912	0.932

EES, *n* (%)	382 (50.7)	819 (51.9)	1446 (49.4)	0.595	0.540	0.111	0.268
BES, *n* (%)	136 (18.0)	283 (17.9)	595 (20.3)	0.954	0.167	0.058	0.099
Others, *n* (%)	66 (8.8)	113 (7.2)	208 (7.1)	0.184	0.139	0.952	0.284
Stent diameter, mm	3.12 ± 0.40	3.17 ± 0.40	3.18 ± 0.42	0.011	<0.001	0.310	0.003
Stent length, mm	30.9± 13.1	29.3 ± 12.4	28.4 ± 11.9	0.004	<0.001	0.015	<0.001
Number of stents	1.19 ± 0.42	1.15 ± 0.38	1.14 ± 0.38	0.052	0.012	0.537	0.026

Values are means ± standard deviation or median (interquartile range) or numbers and percentages. The p Values for continuous data were obtained using analysis of variance or the Jonckheere-Terpstra test for comparisons among three groups, and using the unpaired t-test for comparisons between two groups. The p values for categorical data from chi-square or Fisher's exact test. HFrEF, heart failure with reduced ejection fraction; HFmrEF, heart failure with mildly reduced ejection fraction; HFpEF, heart failure with preserved ejection fraction; NSTEMI, non-ST-segment elevation myocardial infarction; STEMI, ST-segment elevation myocardial infarction; LVEF, left ventricular ejection fraction; SBP, systolic blood pressure; DBP, diastolic blood pressure; CPR, cardiopulmonary resuscitation; SDT, symptom-to-door time; DBT, door-to-balloon time; PCI, percutaneous coronary intervention; CABG, coronary artery bypass graft; CK-MB, creatine kinase myocardial band; HDL, high-density lipoprotein; LDL, low-density lipoprotein; RASI, renin-angiotensin system inhibitor; LAD, left anterior descending artery; LCx, left circumflex artery; RCA, right coronary artery; TIMI, thrombolysis in myocardial infarction; ACC/AHA, American College of Cardiology/American Heart Association; IVUS, intravascular ultrasound; OCT, optical coherence tomography; FFR, fractional flow reserve; ZES, zotarolimus-eluting stent; EES, everolimus-eluting stent; BES, biolimus-eluting stent. [a] Newer-generation drug-eluting stents included in the study were the zotarolimus-eluting stent (Resolute Integrity stent; Medtronic, Inc.), everolimus-eluting stent (Xience Prime stent; Abbott Vascular or Promus Element stent; Boston Scientific), and biolimus-eluting stent (BioMatrix Flex stent; Biosensors International or Nobori stent; Terumo Corporation).

Table S4. Baseline characteristics of the total study population before and after propensity-score matched analysis.

Variables	Entire patients (*n* = 9854)			PSM patients (*n* = 5176)			
	NSTEMI (*n* = 4594)	STEMI (*n* = 5260)	*p*	NSTEMI (*n* = 2588)	STEMI (*n* = 2588)	*p*	SD
Male, n (%)	3335 (72.6)	4145 (78.8)	<0.001	1940 (75.0)	1957 (75.6)	0.606	-0.14
Age, years	64.2 ± 12.0	62.3 ± 12.6	<0.001	63.3 ± 12.2	63.4 ± 12.4	0.818	-0.08
SBP, mmHg	135.6 ± 26.1	127.9 ± 27.8	<0.001	131.9 ± 25.7	132.0 ± 26.8	0.877	-0.04
DBP, mmHg	81.3 ± 15.4	78.3 ± 17.3	<0.001	79.8 ± 15.4	80.0 ± 16.5	0.584	-0.12
Heart rate, beats/min	79.3 ± 17.4	77.3 ± 19.4	<0.001	78.2 ± 17.0	78.4 ± 19.0	0.655	-0.11
Body mass index, Kg/m^2	24.1 ± 3.3	24.1 ± 3.2	0.949	24.0 ± 3.2	24.0 ± 3.1	0.732	0.09
LVEF, %	54.0 ± 10.8	50.3 ± 10.2	<0.001	52.4 ± 10.7	52.3 ± 9.9	0.749	0.09
Killip class II/III, *n* (%)	710 (15.5)	705 (13.4)	0.004	398 (15.4)	401 (15.5)	0.908	0.04
Cardiogenic shock, *n* (%)	84 (1.8)	375 (7.1)	<0.001	74 (2.9)	75 (2.9)	0.934	-0.02
CPR on admission, *n* (%)	142 (3.1)	373 (7.1)	<0.001	112 (4.3)	112 (4.3)	1.000	0
Risk factors							
Hypertension, *n* (%)	2440 (53.1)	2411 (45.8)	<0.001	1271 (49.1)	1249 (48.3)	0.559	0.16
Diabetes mellitus, *n* (%)	1417 (30.8)	1283 (24.4)	<0.001	716 (27.7)	711 (27.5)	0.901	0.04
Dyslipidemia, *n* (%)	557 (12.1)	553 (10.5)	0.013	268 (10.4)	277 (10.7)	0.684	-0.10
Previous MI, *n* (%)	310 (6.7)	240 (4.6)	<0.001	141 (5.4)	152 (5.9)	0.509	-0.22
Previous PCI, *n* (%)	451 (9.8)	320 (6.1)	<0.001	197 (7.6)	206 (8.0)	0.678	-0.15
Previous CABG, *n* (%)	35 (0.8)	18 (0.3)	0.005	18 (0.7)	16 (0.6)	0.864	0.12
Previous stroke, *n* (%)	283 (6,2)	219 (4.2)	<0.001	129 (5.0)	127 (4.9)	0.898	0.05
Current smokers, *n* (%)	1654 (36.0)	2380 (45.2)	<0.001	1403 (40.3)	1051 (40.6)	0.843	-0.06
Laboratory results							
Peak CK-MB, mg/dL	21.4 (6.4–68.3)	156.5 (26.0–300.0)	<0.001	37.8 (8.9–117.3)	50.3 (12.1–134.7)	0.106	-0.20
Peak troponin-I, ng/mL	8.9 (2.5–31.5)	54.4 (16.3–168.8)	<0.001	17.0 (3.4–50.0)	25.0 (7.7–50.0)	0.071	-0.51
Hemoglobin, mg/dL	13.7 ± 2.1	14.2 ± 1.9	<0.001	14.0 ± 2.0	14.0 ± 1.9	0.959	0.02
Blood glucose, mg/dL	159.3 ± 79.2	177.9 ± 79.7	<0.001	166.7 ± 87.4	168.1 ± 71.8	0.545	-0.17
Serum creatinine, mg/dL	1.15 ± 0.67	1.02 ± 0.73	<0.001	1.05 ± 0.95	1.03 ± 0.81	0.387	0.22
Total cholesterol, mg/dL	179.3 ± 45.0	182.6 ± 44.4	<0.001	181.9 ± 43.9	181.1 ± 44.2	0.546	0.18
Triglyceride, mg/dL	132.3 ± 97.5	140.1 ± 92.4	0.001	134.0 ± 98.5	133.9 ± 88.9	0.509	0.10

HDL-cholesterol, mg/dL	42.7 ± 11.6	42.7 ± 12.3	0.765	42.8 ± 11.2	42.7 ± 12.1	0.790	0.08
LDL-cholesterol, mg/dL	113.3 ± 38.8	115.8 ± 39.4	0.003	115.1 ± 36.7	114.9 ± 38.0	0.878	0.05
Discharge medications							
Aspirin, n (%)	4533 (98.7)	5156 (98.0)	0.014	2547 (98.4)	2542 (98.2)	0.666	0.15
Clopidogrel, *n* (%)	3330 (72.5)	3564 (67.8)	<0.001	1806 (69.8)	1807 (69.8)	0.976	-0.01
Ticagrelor, *n* (%)	847 (18.4)	1070 (20.3)	0.018	517 (20.0)	515 (19.9)	0.972	0.02
Prasugrel, *n* (%)	417 (9.1)	626 (11.9)	<0.001	265 (10.2)	266 (10.3)	0.963	-0.03
Beta-blocker, *n* (%)	3879 (84.4)	4511 (85.8)	0.069	2217 (85.7)	2212 (85.5)	0.874	0.06
RASI, *n* (%)	3763 (81.9)	4172 (79.3)	0.001	2116 (81.8)	2099 (81.1)	0.567	0.18
Statin, *n* (%)	4328 (94.2)	4864 (92.5)	0.001	2431 (93.9)	2418 (93.4)	0.493	0.20
Anticoagulant, *n* (%)	87 (1.9)	144 (2.7)	0.006	57 (2.2)	60 (2.3)	0.852	-0.06
Infarct-related artery							
Left main, *n* (%)	143 (3.1)	75 (1.4)	<0.001	41 (1.6)	42 (1.6)	0.913	-0.03
LAD, *n* (%)	1965 (42.8)	2742 (52.1)	<0.001	1231 (47.6)	1264 (48.8)	0.359	-0.24
LCx, *n* (%)	1168 (25.4)	457 (8.7)	<0.001	365 (14.1)	374 (14.5)	0.751	-0.11
RCA, *n* (%)	1318 (28.7)	1986 (37.8)	<0.001	951 (36.7)	908 (35.1)	0.213	0.33
Treated vessel							
Left main, *n* (%)	220 (4.8)	115 (2.2)	<0.001	69 (2.7)	72 (2.8)	0.864	-0.07
LAD, *n* (%)	2658 (57.9)	3202 (60.9)	0.002	1515 (58.5)	1536 (59.4)	0.553	-0.18
LCx, *n* (%)	1777 (38.7)	908 (17.3)	<0.001	662 (25.6)	674 (26.0)	0.727	-0.11
RCA, *n* (%)	1735 (37.9)	2268 (43.1)	<0.001	1101 (42.5)	1082 (41.8)	0.612	0.14
Multivessel disease, *n* (%)	2570 (55.9)	2498 (47.5)	<0.001	1320 (51.0)	1340 (51.8)	0.597	-0.16
Transradial approach, *n* (%)	2361 (51.4)	1368 (26.0)	<0.001	660 (25.5)	655 (25.3)	0.898	0.04
Pre-PCI TIMI flow grade 0/1, *n* (%)	1796 (39.1)	3896 (74.1)	<0.001	1147 (44.3)	1211 (46.8)	0.079	-0.50
ACC/AHA type B2/C lesion, *n* (%)	3883 (84.9)	4569 (88.6)	<0.001	2212 (85.5)	2221 (85.8)	0.721	-0.09
Glycoprotein IIb/IIIa inhibitor, *n* (%)	404 (8.8)	1103 (21.0)	<0.001	287 (11.1)	331 (12.8)	0.065	-0.52
IVUS/OCT, *n* (%)	1169 (25.4)	1049 (19.9)	<0.001	575 (22.2)	581 (22.4)	0.867	-0.04
FFR, *n* (%)	105 (2.3)	38 (0.7)	<0.001	30 (1.2)	30 (1.2)	1.000	0
Drug-eluting stents [a]							
ZES, *n* (%)	1081 (23.5)	1212 (23.0)	0.566	626 (24.2)	614 (23.7)	0.696	0.12
EES, *n* (%)	2386 (51.9)	2647 (50.3)	0.111	1338 (51.7)	1323 (51.1)	0.677	0.12
BES, *n* (%)	920 (20.0)	1014 (19.3)	0.360	484 (18.7)	507 (19.6)	0.437	-0.23

Others, *n* (%)	207 (4.5)	387 (7.4)	<0.001	140 (5.4)	144 (5.6)	0.855	-0.08
Stent diameter, mm	3.07 ± 0.41	3.17 ± 0.41	<0.001	3.12 ± 0.42	3.11 ± 0.41	0.148	0.24
Stent length, mm	29.6 ± 14.1	29.0± 12.3	0.030	29.7 ± 13.9	29.6 ± 13.1	0.835	0.07
Number of stents	1.20 ± 0.45	1.15 ± 0.38	<0.001	1.18 ± 0.43	1.19 ± 0.42	0.948	-0.02

Values are means ± standard deviation or median (interquartile range) or numbers and percentages. The *p* values for continuous data obtained from the unpaired t-test. The *p* values for categorical data from chi-square or Fisher's exact test. SD, standardized mean difference; NSTEMI, non-ST-segment elevation myocardial infarction; STEMI, ST-segment elevation myocardial infarction; SBP, systolic blood pressure; DBP, diastolic blood pressure; LVEF, left ventricular ejection fraction; CPR, cardiopulmonary resuscitation; PCI, percutaneous coronary intervention; CABG, coronary artery bypass graft; CK-MB, creatine kinase myocardial band; HDL, high-density lipoprotein; LDL, low-density lipoprotein; RASI, renin-angiotensin system inhibitor; LAD, left anterior descending artery; LCx, left circumflex artery; RCA, right coronary artery; TIMI, thrombolysis in myocardial infarction; ACC/AHA, American College of Cardiology/American Heart Association; IVUS, intravascular ultrasound; OCT, optical coherence tomography; FFR, fractional flow reserve; ZES, zotarolimus-eluting stent; EES, everolimus-eluting stent; BES, biolimus-eluting stent. [a] Newer-generation drug-eluting stents included in the study were the zotarolimus-eluting stent (Resolute Integrity stent; Medtronic, Inc.), everolimus-eluting stent (Xience Prime stent; Abbott Vascular or Promus Element stent; Boston Scientific), and biolimus-eluting stent (BioMatrix Flex stent; Biosensors International or Nobori stent; Terumo Corporation).

Table S5. Comparison of 3-year mortality between the STEMI and NSTEMI groups based on LVEF groups after excluding in-hospital mortality

Outcomes	Group A NSTEMI (*n* = 465)	Group B STEMI (*n* = 708)	Log-rank	Unadjusted HR (95% CI)	Unadjusted *p* value	Adjusted [a] HR (95% CI)	Adjusted [a] *p* value
**HFrEF (LVEF ≤40%), *n* = 1173**							
POCO	142 (30.5)	143 (20.2)	<0.001	1.608 (1.275–2.028)	<0.001	1.568 (1.224–2.009)	<0.001
All-cause death	99 (21.3)	76 (10.7)	<0.001	2.100 (1.557–2.832)	<0.001	2.182 (1.587–3.000)	<0.001
Cardiac death	65 (14.0)	58 (8.2)	0.001	1.802 (1.265–2.568)	0.001	1.940 (1.329–2.832)	0.001
Non-cardiac death	34 (7.3)	18 (2.5)	<0.001	3.063 (1.730–5.424)	<0.001	2.934 (1.615–5.331)	<0.001
**HFmrEF (LVEF 41–49%), *n* = 2358**							
Outcomes	Group C NSTEMI (*n* = 797)	Group D STEMI (*n* = 1561)	Log-rank	Unadjusted HR (95% CI)	Unadjusted *p* value	Adjusted [a] HR (95% CI)	Adjusted [a] *p* value
POCO	139 (17.4)	206 (13.2)	0.006	1.352 (1.090–1.676)	0.006	1.282 (1.021–1.609)	0.032
All-cause death	72 (9.0)	76 (4.9)	<0.001	1.893 (1.372–2.614)	<0.001	1.786 (1.269–2.512)	0.001
Cardiac death	38 (4.7)	39 (2.5)	0.003	1.946 (1.245–3.042)	0.003	1.821 (1.128–2.940)	0.014
Non-cardiac death	34 (4.3)	37 (2.4)	0.009	1.838 (1.154–2.928)	0.010	1.772 (1.088–2.884)	0.021
**HFpEF (≥50%), *n* = 6073**							
Outcomes	Group E NSTEMI (*n* = 3260)	Group F STEMI (*n* = 2813)	Log-rank	Unadjusted HR (95% CI)	Unadjusted *p* value	Adjusted [a] HR (95% CI)	Adjusted [a] *p* value
POCO	423 (13.0)	369 (13.1)	0.850	0.987 (0.858–1.134)	0.850	0.988 (0.851–1.146)	0.869
All-cause death	141 (4.3)	123 (4.4)	0.943	0.991 (0.778–1.262)	0.943	0.936 (0.722–1.214)	0.619
Cardiac death	70 (2.1)	73 (2.6)	0.261	0.829 (0.597–1.150)	0.262	0.940 (0.662–1.336)	0.730
Non-cardiac death	71 (2.2)	50 (1.8)	0.265	1.228 (0.855–1.764)	0.265	1.249 (0.846–1.847)	0.264
**Total, *n* = 9604**							
Outcomes	NSTEMI (*n* = 4522)	STEMI (*n* = 5082)	Log-rank	Unadjusted HR (95% CI)	Unadjusted *p* value	Adjusted [a] HR (95% CI)	Adjusted [a] *p* value
POCO	704 (15.6)	718 (14.1)	0.051	1.109 (1.000–1.231)	0.051	1.129 (1.012–1.260)	0.030
All-cause death	312 (6.9)	275 (5.4)	0.002	1.286 (1.094–1.513)	0.002	1.367 (1.151–1.623)	<0.001
Cardiac death	173 (3.8)	170 (3.3)	0.187	1.153 (0.933–1.425)	0.188	1.250 (0.999–1.564)	0.048
Non-cardiac death	139 (3.1)	105 (2.1)	0.002	1.503 (1.166–1.936)	0.002	1.550 (1.186–2.027)	0.001

HFrEF, heart failure with reduced ejection fraction; HFmrEF, heart failure with mildly reduced ejection fraction; HFpEF, heart failure with preserved ejection fraction; NSTEMI, non-ST-segment elevation myocardial infarction; STEMI, ST-segment elevation myocardial infarction; HR, hazard ratio; CI, confidence

interval; POCO, patient oriented- composite outcome; SBP, systolic blood pressure; DBP, diastolic blood pressure; BMI, body mass index; CPR, cardiopulmonary resuscitation; SDT, symptom-to-door time; DBT, door-to-balloon time; DM, diabetes mellitus; PCI, percutaneous coronary intervention; CABG, coronary artery bypass graft; CK-MB, peak creatine kinase myocardial band; HDL, high-density lipoprotein. [a]Adjusted by male sex, age, SBP, DBP, heart rate, BMI, Killip class II/III, cardiogenic shock, CPR on admission, SDT, DBT, hypertension, DM, dyslipidemia, previous MI, previous PCI, previous CABG, previous stroke, current smoker, peak CK-MB, peak troponin-I, hemoglobin, blood glucose, serum creatinine, triglyceride, HDL-cholesterol, and aspirin (Table S1).

Table S6. Comparison of 3-year outcomes among three LVEF subgroups in NSTEMI and STEMI.

NSTEMI (*n* = 4594)							
Variables	Group A HFrEF LVEF ≤40% (*n* = 496)	Group C HFmrEF LVEF 41−49% (**n** = 805)	Group E HFpEF LVEF ≥50% (*n* = 3293)	Log-rank			*p* value
				Group A vs. C	Group A vs. E	Group C vs. E	Group A vs. Group C vs. Group E
POCO	173 (34.9)	147 (18.3)	456 (13.8)	<0.001	<0.001	0.002	<0.001
All-cause death	130 (26.2)	80 (9.9)	174 (5.3)	<0.001	<0.001	<0.001	<0.001
Cardiac death	90 (18.1)	42 (5.2)	95 (2.9)	<0.001	<0.001	0.001	<0.001
Non-cardiac death	40 (8.1)	38 (4.7)	79 (2.4)	0.003	<0.001	<0.001	<0.001
Recurrent MI	30 (7.0)	27 (3.5)	90 (2.8)	0.005	<0.001	0.300	<0.001
Any repeat revascularization	53 (12.8)	68 (8.9)	272 (8.5)	0.034	0.004	0.741	0.196
Hospitalization for HF	52 (11.9)	32 (4.1)	58 (1.8)	<0.001	<0.001	<0.001	<0.001
Stroke	20 (4.9)	18 (2.3)	63 (2.0)	0.021	<0.001	0.515	0.011
STEMI (*n* = 5260)							
Variables	Group B HFrEF LVEF ≤40% (*n* = 754)	Group D HFmrEF LVEF 41−49% (*n* = 1578)	Group F HFpEF LVEF ≥50% (*n* = 2928)	Log-rank			*p* value
				Group B vs. D	Group B vs. F	Group D vs. F	Group B vs. Group D vs. Group F
POCO	189 (25.1)	223 (14.1)	484 (16.5)	<0.001	<0.001	0.023	<0.001
All-cause death	122 (16.2)	93 (5.9)	238 (8.1)	<0.001	<0.001	0.005	<0.001
Cardiac death	94 (12.7)	51 (3.3)	178 (6.1)	<0.001	<0.001	<0.001	<0.001
Non-cardiac death	28 (3.5)	42 (2.9)	60 (2.0)	0.088	0.004	0.241	0.027
Recurrent MI	20 (3.0)	45 (3.0)	71 (2.6)	0.956	0.531	0.464	0.685

Any repeat revascularization	58 (8.7)	127 (8.3)	239 (8.7)	0.781	0.989	0.704	0.914
Hospitalization for HF	74 (10.9)	46 (3.0)	44 (1.6)	<0.001	<0.001	0.002	<0.001
Stroke	20 (3.0)	28 (1.8)	45 (1.6)	0.083	0.020	0.628	0.117

NSTEMI, non-ST-segment elevation myocardial infarction; STEMI, ST-segment elevation myocardial infarction; HFrEF, heart failure with reduced ejection fraction; HFmrEF, heart failure with mildly reduced ejection fraction; HFpEF, heart failure with preserved ejection fraction; HR, hazard ratio; CI, confidence interval; POCO, patient oriented-composite outcome.

Table S7. Comparison of 3-year outcomes among three LVEF subgroups in NSTEMI and STEMI after excluding in-hospital mortality.

NSTEMI (*n* = 4522)							
Variables	Group A HFrEF LVEF ≤40% (*n* = 465)	Group C HFmrEF LVEF 41−49% (*n* = 797)	Group E HFpEF LVEF ≥50% (*n* = 3260)	Log-rank Group A vs. C	Log-rank Group A vs. E	Log-rank Group C vs. E	*p* value Group A vs. Group C vs. Group E
POCO	142 (30.5)	139 (17.4)	423 (13.0)	<0.001	<0.001	0.001	<0.001
All-cause death	99 (21.3)	72 (9.0)	141 (4.3)	<0.001	<0.001	<0.001	<0.001
Cardiac death	65 (14.0)	38 (4.8)	70 (2.1)	<0.001	<0.001	<0.001	<0.001
Non-cardiac death	34 (7.3)	34 (4.3)	71 (2.2)	0.027	<0.001	0.002	<0.001
Recurrent MI	30 (6.5)	27 (3.45)	90 (2.8)	0.016	<0.001	0.345	<0.001
Any repeat revascularization	53 (11.4)	68 (8.5)	272 (8.3)	0.112	0.035	0.887	0.090
Hospitalization for HF	52 (11.2)	32 (4.0)	58 (1.8)	<0.001	<0.001	<0.001	<0.001
Stroke	20 (4.3)	18 (2.3)	63 (1.9)	0.059	0.004	0.572	0.005
STEMI (*n* = 5082)							
Variables	Group B HFrEF LVEF ≤40% (*n* = 708)	Group D HFmrEF LVEF 41−49% (*n* = 1561)	Group F HFpEF LVEF ≥50% (*n* = 2813)	Log-rank Group B vs. D	Log-rank Group B vs. F	Log-rank Group D vs. F	*p* value Group B vs. Group D vs. Group F
POCO	143 (20.2)	206 (13.2)	369 (13.1)	<0.001	<0.001	0.963	<0.001
All-cause death	76 (10.7)	76 (4.9)	123 (4.4)	<0.001	<0.001	0.450	<0.001
Cardiac death	58 (8.2)	39 (2.5)	73 (2.6)	<0.001	<0.001	0.921	<0.001
Non-cardiac death	18 (2.5)	37 (2.4)	50 (1.8)	0.771	0.220	0.177	0.264

Recurrent MI	20 (2.8)	45 (2.9)	71 (2.5)	0.939	0.691	0.492	0.754
Any repeat revascularization	58 (8.2)	127 (8.1)	239 (8.5)	0.964	0.880	0.690	0.907
Hospitalization for HF	74 (10.5)	46 (2.9)	44 (1.6)	<0.001	<0.001	0.003	<0.001
Stroke	20 (2.8)	28 (1.8)	45 (1.6)	0.118	0.041	0.624	0.093

NSTEMI, non-ST-segment elevation myocardial infarction; STEMI, ST-segment elevation myocardial infarction; HFrEF, heart failure with reduced ejection fraction; HFmrEF, heart failure with mildly reduced ejection fraction; HFpEF, heart failure with preserved ejection fraction; HR, hazard ratio; CI, confidence interval; POCO, patient oriented-composite outcome.

Table S8. Independent predictors for POCO and all-cause death in the total study population.

Variables	POCO				All-cause death			
	Unadjusted		Adjusted		Unadjusted		Adjusted	
	HR (95% CI)	*p*	HR (95% CI)	*p*	HR (95% CI)	*p*	HR (95% CI)	*p*
NSTEMI vs. STEMI	1.020 (0.927–1.123)	0.681	1.064 (0.963 – 1.176)	0.224	1.041 (0.908–1.192)	0.567	1.023 (0.887–1.180)	0.750
Male	1.254 (1.137–1.407)	<0.001	1.066 (0.948 – 1.198)	0.285	1.655 (1.443–1.921)	<0.001	1.030 (0.883–1.203)	0.705
Age, ≥65 years	1.931 (1.750–2.132)	<0.001	1.471 (1.316 – 1.643)	<0.001	4.833 (4.076–5.731)	<0.001	3.333 (2.768–4.013)	<0.001
Cardiogenic shock	2.424 (2.051–2.865)	<0.001	1.324 (1.110 – 1.578)	0.002	3.861 (3.173–4.698)	<0.001	1.519 (1.233–1.870)	<0.001
CPR on admission	5.968 (5.245–6.791)	<0.001	3.189 (2.758 – 3.687)	<0.001	11.93 (10.28–13.84)	<0.001	4.429 (3.707–5.291)	<0.001
Hypertension	1.523 (1.382–1.679)	<0.001	1.269 (1.144 – 1.407)	<0.001	1.748 (1.520–2.010)	<0.001	1.255 (1.083–1.454)	0.002
Diabetes mellitus	1.683 (1.524–1.858)	<0.001	1.247 (1.125 – 1.382)	<0.001	2.002 (1.745–2.296)	<0.001	1.299 (1.127–1.499)	<0.001
Current smoker	1.475 (1.332–1.633)	<0.001	1.105 (0.985 – 1.240)	0.087	2.076 (1.777–2.425)	<0.001	1.118 (0.942–1.327)	0.203
Beta-blocker non-use	2.553 (2.292–2.843)	<0.001	1.707 (1.516 – 1.923)	<0.001	4.675 (4.074–5.366)	<0.001	2.133 (1.816–2.505)	<0.001
RASI non-use	2.185 (1.971–2.423)	<0.001	1.529 (1.366 – 1.712)	<0.001	3.780 (3.299–4.332)	<0.001	1.951 (1.669–2.281)	<0.001
Statin non-use	4.832 (4.275–5.461)	<0.001	2.987 (2.614 – 3.414)	<0.001	9.369 (8.104–10.83)	<0.001	3.902 (3.291–4.626)	<0.001
Multivessel disease	1.215 (1.093–1.350)	<0.001	1.675 (1.511 – 1.857)	<0.001	1.674 (1.454–1.927)	<0.001	1.211 (1.049–1.398)	0.009
Pre-PCI TIMI flow grade 0/1	1.125 (1.022–1.239)	0.016	1.076 (0.976 – 1.186)	0.143	1.004 (0.876–1.152)	0.950	1.042 (0.906–1.197)	0.562

POCO, patient-oriented composite outcome; NSTEMI, non-ST-segment elevation myocardial infarction; STEMI, ST-segment elevation myocardial infarction; CPR, cardiopulmonary resuscitation; RASI, renin-angiotensin system inhibitor; PCI, percutaneous coronary intervention; TIMI, thrombolysis in myocardial infarction.

Table S9. Independent predictors for POCO and all-cause death among patients excluding in-hospital mortality.

Variables	POCO				All-cause death			
	Unadjusted		Adjusted		Unadjusted		Adjusted	
	HR (95% CI)	*p*	HR (95% CI)	*p*	HR (95% CI)	*p*	HR (95% CI)	*p*
NSTEMI vs. STEMI	1.109 (1.000–1.231)	0.051	1.141 (1.031 – 1.285)	0.027	1.286 (1.094–1.513)	0.001	1.315 (1.125–1.695)	<0.001
Male	1.222 (1.088–1.374)	0.001	1.089 (0.958 – 1.239)	0.191	1.730 (1.460–2.051)	<0.001	1.038 (0.864–1.247)	0.692
Age, ≥65 years	1.745 (1.570–1.940)	<0.001	1.469 (1.304 – 1.654)	<0.001	5.420 (4.395–6.684)	<0.001	4.595 (3.664–5.761)	<0.001
Cardiogenic shock	1.410 (1.119–1.776)	0.004	1.165 (0.920 – 1.476)	0.206	1.876 (1.366–2.577)	<0.001	1.362 (0.984–1.884)	0.062
CPR on admission	1.845 (1.466–2.321)	<0.001	1.409 (1.112 – 1.787)	0.005	2.871 (2.143–3.846)	<0.001	1.924 (1.419–2.609)	<0.001
Hypertension	1.522 (1.370–1.691)	<0.001	1.247 (1.115 – 1.395)	<0.001	1.853 (1.566–2.192)	<0.001	1.234 (1.034–1.474)	0.020
Diabetes mellitus	1.612 (1.447–1.796)	<0.001	1.337 (1.195 – 1.495)	<0.001	1.948 (1.653–2.296)	<0.001	1.500 (1.265–1.777)	<0.001
Current smoker	1.392 (1.248–1.553)	<0.001	1.093 (0.965 – 1.237)	0.162	2.058 (1.711–2.476)	<0.001	1.073 (0.874–1.316)	0.502
Beta-blocker non-use	1.369 (1.190–1.575)	<0.001	1.240 (1.072 – 1.436)	0.004	1.832 (1.501–2.234)	<0.001	1.439 (1.168–1.772)	0.001
RASI non-use	1.260 (1.109–1.432)	<0.001	1.164 (1.018 – 1.330)	0.026	1.529 (1.266–1.847)	<0.001	1.306 (1.072–1.591)	0.008
Statin non-use	1.789 (1.471–2.177)	<0.001	1.563 (1.280 – 1.908)	<0.001	2.516 (1.934–3.273)	<0.001	1.900 (1.450–2.489)	<0.001
Multivessel disease	1.949 (1.747–2.175)	<0.001	1.752 (1.568 – 1.957)	<0.001	1.516 (1.285–1.799)	<0.001	1.197 (1.012–1.415)	0.036
Pre-PCI TIMI flow grade 0/1	1.247 (1.123–1.383)	<0.001	1.154 (1.039 – 1.283)	0.007	1.228 (1.045–1.444)	0.013	1.312 (1.104–1.529)	0.010

POCO, patient-oriented composite outcome; NSTEMI, non-ST-segment elevation myocardial infarction; STEMI, ST-segment elevation myocardial infarction; CPR, cardiopulmonary resuscitation; RASI, renin-angiotensin system inhibitor; PCI, percutaneous coronary intervention; TIMI, thrombolysis in myocardial infarction.

Table S10. In-hospital mortality between the STEMI and NSTEMI groups across three LVEF subgroups in male patients.

Outcomes	Group A1 NSTEMI (n = 326)	Group A2 STEMI (n = 567)	Log-rank	Unadjusted HR (95% CI)	Unadjusted *p* value	Adjusted [a] HR (95% CI)	Adjusted [a] *p* value
HFrEF (LVEF ≤40%), *n* = 893							
All-cause death	22 (6.8)	30 (5.3)	0.350	1.299 (0.749–2.252)	0.352	0.998 (0.992–1.003)	0.387
Cardiac death	18 (5.7)	24 (4.2)	0.362	1.327 (0.720–2.445)	0.365	1.677 (0.834–3.373)	0.147
Non-cardiac death	4 (1.1)	6 (1.2)	0.851	0.932 (0.238–2.986)	0.847	0.963 (0.236–3.921)	0.958
HFmrEF (LVEF 41–49%), *n* = 1789							
Outcomes	Group B1 NSTEMI (n = 557)	Group B2 STEMI (n = 1232)	Log-rank	Unadjusted HR (95% CI)	Unadjusted *p* value	Adjusted [a] HR (95% CI)	Adjusted [a] *p* value
All-cause death	6 (1.2)	11 (0.9)	0.685	1.208 (0.447–3.265)	0.695	1.366 (0.477–3.909)	0.561
Cardiac death	3 (0.6)	8 (0.6)	0.782	0.929 (0.220–3.126)	0.782	0.860 (0.208–3.564)	0.836
Non-cardiac death	3 (0.6)	3 (0.3)	0.316	2.218 (0.448–10.99)	0.329	1.862 (0.342–10.13)	0.472
HFpEF (≥50%), *n* = 4798							
Outcomes	Group C1 NSTEMI (n = 2452)	Group C2 STEMI (n = 2346)	Log-rank	Unadjusted HR (95% CI)	Unadjusted *p* value	Adjusted [a] HR (95% CI)	Adjusted [a] *p* value
All-cause death	23 (0.9)	77 (3.3)	<0.001	0.282 (0.177–0.450)	<0.001	0.345 (0.211–0.563)	<0.001
Cardiac death	16 (0.7)	71 (3.0)	<0.001	0.213 (0.124–0.367)	<0.001	0.255 (0.145–0.451)	<0.001
Non-cardiac death	7 (0.2)	6 (0.3)	0.876	0.714 (0.308–2.728)	0.876	0.662 (0.208–2.107)	0.485
Total (*n* = 7480)							
Outcomes	NSTEMI (n = 3335)	STEMI (n = 4145)	Log-rank	Unadjusted HR (95% CI)	Unadjusted *p* value	Adjusted [a] HR (95% CI)	Adjusted [a] *p* value
All-cause death	51 (1.5)	118 (2.8)	<0.001	0.533 (0.384–0.740)	<0.001	0.583 (0.414–0.820)	0.002
Cardiac death	37 (1.1)	103 (2.4)	<0.001	0.443 (0.305–0.646)	<0.001	0.488 (0.330–0.722)	<0.001
Non-cardiac death	14 (0.4)	15 (0.4)	0.713	1.046 (0.553–2.375)	0.713	1.199 (0.563–2.553)	0.638

HFrEF, heart failure with reduced ejection fraction; HFmrEF, heart failure with mildly reduced ejection fraction; HFpEF, heart failure with preserved ejection fraction; NSTEMI, non-ST-segment elevation myocardial infarction; STEMI, ST-segment elevation myocardial infarction; HR, hazard ratio; CI, confidence interval; SBP, systolic blood pressure; DBP, diastolic blood pressure; BMI, body mass index; CPR, cardiopulmonary resuscitation; SDT, symptom-to-door time; DBT, door-to-balloon time; DM, diabetes mellitus; PCI, percutaneous coronary intervention; CABG, coronary artery bypass graft; CK-MB, peak creatine kinase

myocardial band; HDL, high-density lipoprotein. [a] Adjusted by male sex, age, SBP, DBP, heart rate, BMI, Killip class II/III, cardiogenic shock, CPR on admission, SDT, DBT, hypertension, DM, dyslipidemia, previous MI, previous PCI, previous CABG, previous stroke, current smoker, peak CK-MB, peak troponin-I, hemoglobin, blood glucose, serum creatinine, triglyceride, HDL-cholesterol, and aspirin (Table S1).

Table S11. In-hospital mortality between the STEMI and NSTEMI groups across three LVEF subgroups in female patients.

			HFrEF (LVEF ≤40%), *n* = 357				
Outcomes	Group D1 NSTEMI (n = 170)	Group D2 STEMI (n = 187)	Log-rank	Unadjusted HR (95% CI)	*p* value	Adjusted [a] HR (95% CI)	*p* value
All-cause death	9 (5.3)	16 (8.6)	0.222	0.605 (0.267–1.369)	0.228	0.600 (0.245–1.357)	0.214
Cardiac death	7 (4.1)	12 (6.5)	0.322	0.628 (0.247–1.594)	0.327	0.654 (0.255–1.820)	0.416
Non-cardiac death	2 (1.2)	4 (2.1)	0.465	0.537 (0.098–2.930)	0.472	0.380 (0.059–2.450)	0.309
			HFmrEF (LVEF 41–49%), *n* = 594				
Outcomes	Group E1 NSTEMI (n = 248)	Group E2 STEMI (n = 346)	Log-rank	Unadjusted HR (95% CI)	*p* value	Adjusted [a] HR (95% CI)	*p* value
All-cause death	2 (0.8)	6 (1.7)	0.334	0.463 (0.094–2.296)	0.337	0.436 (0.081–2.258)	0.331
Cardiac death	1 (0.4)	4 (1.2)	0.325	0.349 (0.039–3.118)	0.323	0.307 (0.030–3.104)	0.302
Non-cardiac death	1 (0.4)	2 (0.5)	0.762	0.692 (0.063–7.631)	0.764	0.640 (0.049–8.323)	0.733
			HFpEF (≥50%), *n* = 1423				
Outcomes	Group F1 NSTEMI (n = 841)	Group F2 STEMI (n = 582)	Log-rank	Unadjusted HR (95% CI)	*p* value	Adjusted [a] HR (95% CI)	*p* value
All-cause death	10 (1.2)	38 (6.5)	<0.001	0.177 (0.088–0.356)	<0.001	0.166 (0.080–0.344)	<0.001
Cardiac death	9 (1.1)	34 (5.8)	<0.001	0.179 (0.086–0.373)	<0.001	0.172 (0.075–0.335)	<0.001
Non-cardiac death	1 (0.1)	4 (0.7)	0.098	0.164 (0.018–1.468)	0.106	0.157 (0.016–1.455)	0.102
			Total (*n* = 2374)				
Outcomes	NSTEMI (n = 1259)	STEMI (n = 1115)	Log-rank	Unadjusted HR (95% CI)	*p* value	Adjusted [a] HR (95% CI)	*p* value
All-cause death	21 (1.7)	60 (5.4)	<0.001	0.303 (0.185–0.499)	<0.001	0.304 (0.181–0.510)	<0.001
Cardiac death	17 (1.4)	50 (4.5)	<0.001	0.296 (0.171–0.513)	<0.001	0.306 (0.172–0.542)	<0.001
1Non-cardiac death	4 (0.3)	10 (0.9)	0.057	0.342 (0.107–1.090)	0.070	0.287 (0.084–0.980)	0.052

HFrEF, heart failure with reduced ejection fraction; HFmrEF, heart failure with mildly reduced ejection fraction; HFpEF, heart failure with preserved ejection fraction; NSTEMI, non-ST-segment elevation myocardial infarction; STEMI, ST-segment elevation myocardial infarction; HR, hazard ratio; CI, confidence interval; SBP, systolic blood pressure; DBP, diastolic blood pressure; BMI, body mass index; CPR, cardiopulmonary resuscitation; SDT, symptom-to-door time; DBT, door-to-balloon time; DM, diabetes mellitus; PCI, percutaneous coronary intervention; CABG, coronary artery bypass graft; CK-MB, peak creatine kinase myocardial band; HDL, high-density lipoprotein. [a] Adjusted by male sex, age, SBP, DBP, heart rate, BMI, Killip class II/III, cardiogenic shock, CPR on admission, SDT, DBT, hypertension, DM, dyslipidemia, previous MI, previous PCI, previous CABG, previous stroke, current smoker, peak CK-MB, peak troponin-I, hemoglobin, blood glucose, serum creatinine, triglyceride, HDL-cholesterol, and aspirin (Table S1).

Table S12. Comparison of 3-year mortality between the STEMI and NSTEMI groups based on LVEF groups in male patients

			HFrEF (LVEF ≤40%), *n* = 893				
Outcomes	Group A1 NSTEMI (*n* = 326)	Group A2 STEMI (*n* = 567)	Log-rank	Unadjusted HR (95% CI)	Unadjusted *p* value	Adjusted [a] HR (95% CI)	Adjusted [a] *p* value
POCO	112 (34.4)	135 (23.8)	0.001	1.543 (1.201–1.982)	0.001	1.604 (1.226–2.098)	0.001
All-cause death	85 (26.1)	78 (13.8)	<0.001	2.013 (1.480–2.737)	<0.001	2.256 (1.615–3.153)	<0.001
Cardiac death	60 (18.4)	59 (10.4)	0.001	1.871 (1.306–2.680)	0.001	2.175 (1.465–3.231)	<0.001
Non-cardiac death	25 (7.7)	19 (3.4)	0.002	2.456 (1.353–4.461)	0.003	2.523 (1.341–4.745)	0.004
			HFmrEF (LVEF 41–49%), *n* = 1789				
Outcomes	Group B1 NSTEMI (*n* = 557)	Group B2 STEMI (*n* = 1232)	Log-rank	Unadjusted HR (95% CI)	Unadjusted *p* value	Adjusted [a] HR (95% CI)	Adjusted [a] *p* value
POCO	96 (17.2)	165 (13.4)	0.033	1.314 (1.022–1.690)	0.033	1.310 (1.004–1.709)	0.046
All-cause death	53 (9.5)	63 (5.1)	<0.001	1.898 (1.317–2.735)	0.001	1.967 (1.333–2.903)	0.001
Cardiac death	30 (5.4)	33 (2.7)	0.004	2.049 (1.250–3.359)	0.004	2.110 (1.239–3.592)	0.006
Non-cardiac death	23 (4.1)	30 (2.4)	0.045	1.732 (1.006–2.982)	0.047	1.827 (1.031–3.238)	0.039
			HFpEF (≥50%), *n* = 4798				
Outcomes	Group C1 NSTEMI (*n* = 2452)	Group C2 STEMI (*n* = 2346)	Log-rank	Unadjusted HR (95% CI)	Unadjusted *p* value	Adjusted [a] HR (95% CI)	Adjusted [a] *p* value
POCO	332 (13.5)	362 (15.4)	0.044	0.858 (0.739–0.996)	0.044	0.955 (0.812–1.123)	0.578
All-cause death	110 (4.6)	163 (6.9)	<0.001	0.634 (0.498–0.807)	<0.001	0.766 (0.585–1.002)	0.048
Cardiac death	55 (2.3)	121 (5.1)	<0.001	0.428 (0.311–0.588)	<0.001	0.566 (0.397–0.807)	0.002
Non-cardiac death	55 (2.3)	42 (1.8)	0.319	1.226 (0.821–1.832)	0.320	1.238 (0.801–1.912)	0.336

Outcomes	Total, n = 7480						
	NSTEMI (n = 3335)	STEMI (n = 4145)	Log-rank	Unadjusted HR (95% CI)	p value	Adjusted [a] HR (95% CI)	p value
POCO	540 (16.2)	662 (16.0)	0.897	1.008 (0.899–1.129)	0.897	1.113 (0.986–1.257)	0.084
All-cause death	248 (7.4)	304 (7.3)	0.918	1.009 (0.853–1.193)	0.918	1.236 (1.032–1.481)	0.021
Cardiac death	145 (4.3)	213 (5.2)	0.108	0.841 (0.681–1.039)	0.109	0.918 (0.731–1.153)	0.462
Non-cardiac death	103 (3.1)	91 (2.1)	0.019	1.401 (1.057–1.857)	0.019	1.524 (1.130–2.055)	0.006

HFrEF, heart failure with reduced ejection fraction; HFmrEF, heart failure with mildly reduced ejection fraction; HFpEF, heart failure with preserved ejection fraction; NSTEMI, non-ST-segment elevation myocardial infarction; STEMI, ST-segment elevation myocardial infarction; HR, hazard ratio; CI, confidence interval; POCO, patient oriented- composite outcome; SBP, systolic blood pressure; DBP, diastolic blood pressure; BMI, body mass index; CPR, cardiopulmonary resuscitation; SDT, symptom-to-door time; DBT, door-to-balloon time; DM, diabetes mellitus; PCI, percutaneous coronary intervention; CABG, coronary artery bypass graft; CK-MB, peak creatine kinase myocardial band; HDL, high-density lipoprotein. [a]Adjusted by male sex, age, SBP, DBP, heart rate, BMI, Killip class II/III, cardiogenic shock, CPR on admission, SDT, DBT, hypertension, DM, dyslipidemia, previous MI, previous PCI, previous CABG, previous stroke, current smoker, peak CK-MB, peak troponin-I, hemoglobin, blood glucose, serum creatinine, triglyceride, HDL-cholesterol, and aspirin (Table S1).

Table S13. Comparison of 3-year mortality between the STEMI and NSTEMI groups based on LVEF groups in female patients

Outcomes	HFrEF (LVEF ≤40%), n = 357						
	Group D1 NSTEMI (n = 170)	Group D2 STEMI (n = 187)	Log-rank	Unadjusted HR (95% CI)	p value	Adjusted [a] HR (95% CI)	p value
POCO	61 (35.9)	54 (28.9)	0.227	1.252 (0.868–1.807)	0.228	1.238 (0.798–1.694)	0.432
All-cause death	45 (26.5)	44 (23.5)	0.616	1.112 (0.734–1.685)	0.617	1.093 (0.715–1.671)	0.681
Cardiac death	30 (17.7)	35 (18.9)	0.773	0.931 (0.572–1.516)	0.773	0.923 (0.560–1.511)	0.752
Non-cardiac death	15 (8.8)	9 (4.6)	0.150	1.818 (0.795–4.454)	0.156	1.811 (0.782–4.194)	0.176
	HFmrEF (LVEF 41–49%), n = 594						
Outcomes	Group E1 NSTEMI (n = 248)	Group E2 STEMI (n = 346)	Log-rank	Unadjusted HR (95% CI)	p value	Adjusted [a] HR (95% CI)	p value
MuPOCO	51 (20.6)	58 (16.8)	0.267	1.237 (0.849–1.802)	0.268	1.208 (0.826–1.767)	0.290
All-cause death	27 (10.9)	30 (8.7)	0.387	1.257 (0.748–2.115)	0.388	1.175 (0.693–1.992)	0.449
Cardiac death	12 (4.8)	18 (5.2)	0.847	0.930 (0.448–1.932)	0.847	0.872 (0.416–1.831)	0.718
Non-cardiac death	15 (6.1)	12 (3.5)	0.144	1.749 (0.818–3.736)	0.149	1.628 (0.754–3.514)	0.194
	HFpEF (≥50%), n = 1423						

Outcomes	Group F1 NSTEMI (*n* = 841)	Group F2 STEMI (*n* = 582)	Log-rank	Unadjusted		Adjusted [a]	
				HR (95% CI)	*p* value	HR (95% CI)	*p* value
POCO	124 (14.7)	122 (21.0)	0.001	0.662 (0.515–0.850)	0.001	0.637 (0.493–0.822)	0.001
All-cause death	64 (7.6)	75 (12.9)	0.001	0.564 (0.404–0.787)	0.001	0.551 (0.390–0.780)	0.001
Cardiac death	40 (4.8)	57 (9.8)	<0.001	0.466 (0.311–0.698)	<0.001	0.448 (0.292–0.688)	<0.001
1Non-cardiac death	24 (2.8)	18 (3.1)	0.659	0.871 (0.473–1.606)	0.659	0.883 (0.476–1.638)	0.693
				Total, *n* = 2374			
Outcomes	NSTEMI (*n* = 1259)	STEMI (*n* = 1115)	Log-rank	Unadjusted		Adjusted [a]	
				HR (95% CI)	*p* value	HR (95% CI)	*p* value
POCO	236 (18.7)	234 (21.0)	0.106	0.862 (0.719–1.032)	0.106	0.879 (0.727–1.072)	0.209
All-cause death	136 (10.8)	149 (13.4)	0.038	0.783 (0.620–0.988)	0.039	0.753 (0.594–0.955)	0.019
Cardiac death	82 (6.5)	110 (9.9)	0.002	0.641 (0.481–0.853)	0.002	0.607 (0.453–0.815)	0.001
Non-cardiac death	54 (4.3)	39 (3.5)	0.424	1.183 (0.784–1.786)	0.424	1.172 (0.773–1.776)	0.455

HFrEF, heart failure with reduced ejection fraction; HFmrEF, heart failure with mildly reduced ejection fraction; HFpEF, heart failure with preserved ejection fraction; NSTEMI, non-ST-segment elevation myocardial infarction; STEMI, ST-segment elevation myocardial infarction; HR, hazard ratio; CI, confidence interval; POCO, patient oriented- composite outcome; SBP, systolic blood pressure; DBP, diastolic blood pressure; BMI, body mass index; CPR, cardiopulmonary resuscitation; SDT, symptom-to-door time; DBT, door-to-balloon time; DM, diabetes mellitus; PCI, percutaneous coronary intervention; CABG, coronary artery bypass graft; CK-MB, peak creatine kinase myocardial band; HDL, high-density lipoprotein. [a]Adjusted by male sex, age, SBP, DBP, heart rate, BMI, Killip class II/III, cardiogenic shock, CPR on admission, SDT, DBT, hypertension, DM, dyslipidemia, previous MI, previous PCI, previous CABG, previous stroke, current smoker, peak CK-MB, peak troponin-I, hemoglobin, blood glucose, serum creatinine, triglyceride, HDL-cholesterol, and aspirin (Table S1).

Table S14. Comparison of 3-year mortality between the STEMI and NSTEMI groups based on LVEF subgroups after excluding in-hospital mortality in male patients

	HFrEF (LVEF ≤40%), *n* = 841						
Outcomes	Group A1 NSTEMI (*n* = 304)	Group A2 STEMI (*n* = 537)	Log-rank	Unadjusted HR (95% CI)	Unadjusted *p* value	Adjusted [a] HR (95% CI)	Adjusted [a] *p* value
POCO	90 (29.6)	105 (19.6)	0.001	1.615 (1.218–2.140)	0.001	1.466 (1.075–1.999)	0.016
All-cause death	63 (20.7)	48 (8.9)	<0.001	2.473 (1.699–3.600)	<0.001	2.244 (1.479–3.404)	<0.001
Cardiac death	42 (13.8)	35 (6.5)	<0.001	2.257 (1.441–3.535)	<0.001	1.936 (1.181–3.175)	0.009
Non-cardiac death	21 (6.9)	13 (2.4)	0.001	3.057 (1.531–6.106)	0.002	3.206 (1.571–6.987)	0.001
	HFmrEF (LVEF 41–49%), *n* = 1772						
Outcomes	Group B1 NSTEMI (*n* = 551)	Group B2 STEMI (*n* = 1221)	Log-rank	Unadjusted HR (95% CI)	Unadjusted *p* value	Adjusted [a] HR (95% CI)	Adjusted [a] *p* value
POCO	90 (16.3)	154 (12.6)	0.034	1.323 (1.020–1.715)	0.035	1.294 (0.985–1.700)	0.048
All-cause death	47 (8.5)	52 (4.3)	<0.001	2.047 (1.379–3.036)	<0.001	1.759 (1.159–2.669)	0.008
Cardiac death	27 (4.9)	25 (2.1)	0.001	2.445 (1.419–4.212)	0.001	1.862 (1.052–3.295)	0.033
Non-cardiac death	20 (3.6)	27 (2.2)	0.076	1.678 (0.941–2.992)	0.079	1.656 (0.897–2.847)	0.107
	HFpEF (≥50%), *n* = 4698						
Outcomes	Group C1 NSTEMI (*n* = 2429)	Group C2 STEMI (*n* = 2269)	Log-rank	Unadjusted HR (95% CI)	Unadjusted *p* value	Adjusted [a] HR (95% CI)	Adjusted [a] *p* value
POCO	309 (12.7)	285 (12.6)	0.878	1.013 (0.862–1.190)	0.878	1.054 (0.888–1.251)	0.548
All-cause death	87 (3.6)	86 (3.8)	0.721	0.947 (0.703–1.276)	0.721	0.922 (0.673–1.261)	0.610
Cardiac death	39 (1.6)	50 (2.2)	0.139	0.730 (0.480–1.110)	0.141	0.919 (0.595–1.431)	0.923
Non-cardiac death	48 (2.0)	36 (1.6)	0.313	1.249 (0.811–1.924)	0.314	1.233 (0.778–1.916)	0.373
	Total, *n* = 7311						
Outcomes	NSTEMI (*n* = 3284)	STEMI (*n* = 4027)	Log-rank	Unadjusted HR (95% CI)	Unadjusted *p* value	Adjusted [a] HR (95% CI)	Adjusted [a] *p* value
POCO	489 (14.9)	544 (13.5)	0.092	1.111 (0.983–1.255)	0.092	1.139 (1.001–1.297)	0.052
All-cause death	197 (6.0)	186 (4.6)	0.008	1.311 (1.073–1.602)	0.008	1.392 (1.123–1.724)	0.002
Cardiac death	108 (3.3)	110 (2.7)	0.151	1.214 (0.931–1.584)	0.152	1.318 (0.992–1.750)	0.057
Non-cardiac death	89 (2.7)	76 (1.9)	0.016	1.451 (1.069–1.971)	0.017	1.490 (1.076–2.063)	0.016

HFrEF, heart failure with reduced ejection fraction; HFmrEF, heart failure with mildly reduced ejection fraction; HFpEF, heart failure with preserved ejection

fraction; NSTEMI, non-ST-segment elevation myocardial infarction; STEMI, ST-segment elevation myocardial infarction; HR, hazard ratio; CI, confidence interval; POCO, patient oriented- composite outcome; SBP, systolic blood pressure; DBP, diastolic blood pressure; BMI, body mass index; CPR, cardiopulmonary resuscitation; SDT, symptom-to-door time; DBT, door-to-balloon time; DM, diabetes mellitus; PCI, percutaneous coronary intervention; CABG, coronary artery bypass graft; CK-MB, peak creatine kinase myocardial band; HDL, high-density lipoprotein. [a]Adjusted by male sex, age, SBP, DBP, heart rate, BMI, Killip class II/III, cardiogenic shock, CPR on admission, SDT, DBT, hypertension, DM, dyslipidemia, previous MI, previous PCI, previous CABG, previous stroke, current smoker, peak CK-MB, peak troponin-I, hemoglobin, blood glucose, serum creatinine, triglyceride, HDL-cholesterol, and aspirin (Table S1).

Table S15. Comparison of 3-year mortality between the STEMI and NSTEMI groups based on LVEF subgroups after excluding in-hospital mortality in female patients

	HFrEF (LVEF ≤40%), *n* = 332						
Outcomes	Group D1 NSTEMI (*n* = 161)	Group D2 STEMI (*n* = 171)	Log-rank	Unadjusted HR (95% CI)	Unadjusted *p* value	Adjusted [a] HR (95% CI)	Adjusted [a] *p* value
POCO	52 (32.3)	38 (22.2)	0.045	1.529 (1.006–2.324)	0.047	1.349 (0.880–2.069)	0.170
All-cause death	36 (22.4)	28 (16.4)	0.176	1.404 (0.857–2.301)	0.178	1.302 (0.786–2.156)	0.306
Cardiac death	23 (14.3)	23 (13.5)	0.770	1.090 (0.611–1.943)	0.771	1.043 (0.576–1.888)	0.890
Non-cardiac death	13 (8.1)	5 (2.9)	0.037	2.852 (1.017–8.001)	0.046	2.764 (0.958–7.974)	0.060
	HFmrEF (LVEF 41–49%), *n* = 586						
Outcomes	Group E1 NSTEMI (*n* = 246)	Group E2 STEMI (*n* = 340)	Log-rank	Unadjusted HR (95% CI)	Unadjusted *p* value	Adjusted [a] HR (95% CI)	Adjusted [a] *p* value
POCO	49 (19.9)	52 (15.3)	0.154	1.327 (0.898–1.960)	0.155	1.177 (0.790–1.754)	0.424
All-cause death	25 (10.2)	24 (7.1)	0.186	1.456 (0.832–2.550)	0.188	1.181 (0.664–2.100)	0.571
Cardiac death	11 (4.5)	14 (4.1)	0.819	1.096 (0.498–2.415)	0.819	1.016 (0.456–2.266)	0.969
Non-cardiac death	14 (5.7)	10 (3.0)	0.097	1.901 (0.871–4.414)	0.098	1.394 (0.596–3.262)	0.444
	HFpEF (≥50%), *n* = 1375						
Outcomes	Group F1 NSTEMI (*n* = 831)	Group F2 STEMI (*n* = 544)	Log-rank	Unadjusted HR (95% CI)	Unadjusted *p* value	Adjusted [a] HR (95% CI)	Adjusted [a] *p* value
POCO	114 (13.7)	84 (15.4)	0.356	0.876 (0.661–1.161)	0.357	0.807 (0.604–1.080)	0.149
All-cause death	54 (6.5)	37 (6.8)	0.822	0.953 (0.627–1.448)	0.822	0.851 (0.552–1.313)	0.466
Cardiac death	31 (3.7)	23 (4.2)	0.643	0.880 (0.513–1.510)	0.644	0.748 (0.424–1.317)	0.314
Non-cardiac death	23 (2.8)	14 (2.6)	0.836	1.073 (0.552–2.084)	0.836	1.025 (0.521–2.018)	0.943

Outcomes	Total, n = 2293						
	NSTEMI (n = 1238)	STEMI (n = 1055)	Log-rank	Unadjusted HR (95% CI)	p value	Adjusted [a] HR (95% CI)	p value
POCO	215 (17.4)	174 (16.5)	0.620	1.052 (0.861–1.285)	0.620	1.049 (0.857–1.285)	0.642
All-cause death	115 (9.3)	89 (8.4)	0.488	1.103 (0.836–1.454)	0.488	1.036 (0.782–1.372)	0.806
Cardiac death	65 (5.3)	60 (5.7)	0.660	0.924 (0.651–1.313)	0.660	0.813 (0.569–1.162)	0.257
Non-cardiac death	50 (4.0)	29 (2.7)	0.095	1.472 (0.932–2.327)	0.097	1.270 (0.798–2.022)	0.313

HFrEF, heart failure with reduced ejection fraction; HFmrEF, heart failure with mildly reduced ejection fraction; HFpEF, heart failure with preserved ejection fraction; NSTEMI, non-ST-segment elevation myocardial infarction; STEMI, ST-segment elevation myocardial infarction; HR, hazard ratio; CI, confidence interval; POCO, patient oriented- composite outcome; SBP, systolic blood pressure; DBP, diastolic blood pressure; BMI, body mass index; CPR, cardiopulmonary resuscitation; SDT, symptom-to-door time; DBT, door-to-balloon time; DM, diabetes mellitus; PCI, percutaneous coronary intervention; CABG, coronary artery bypass graft; CK-MB, peak creatine kinase myocardial band; HDL, high-density lipoprotein. [a]Adjusted by male sex, age, SBP, DBP, heart rate, BMI, Killip class II/III, cardiogenic shock, CPR on admission, SDT, DBT, hypertension, DM, dyslipidemia, previous MI, previous PCI, previous CABG, previous stroke, current smoker, peak CK-MB, peak troponin-I, hemoglobin, blood glucose, serum creatinine, triglyceride, HDL-cholesterol, and aspirin (Table S1).

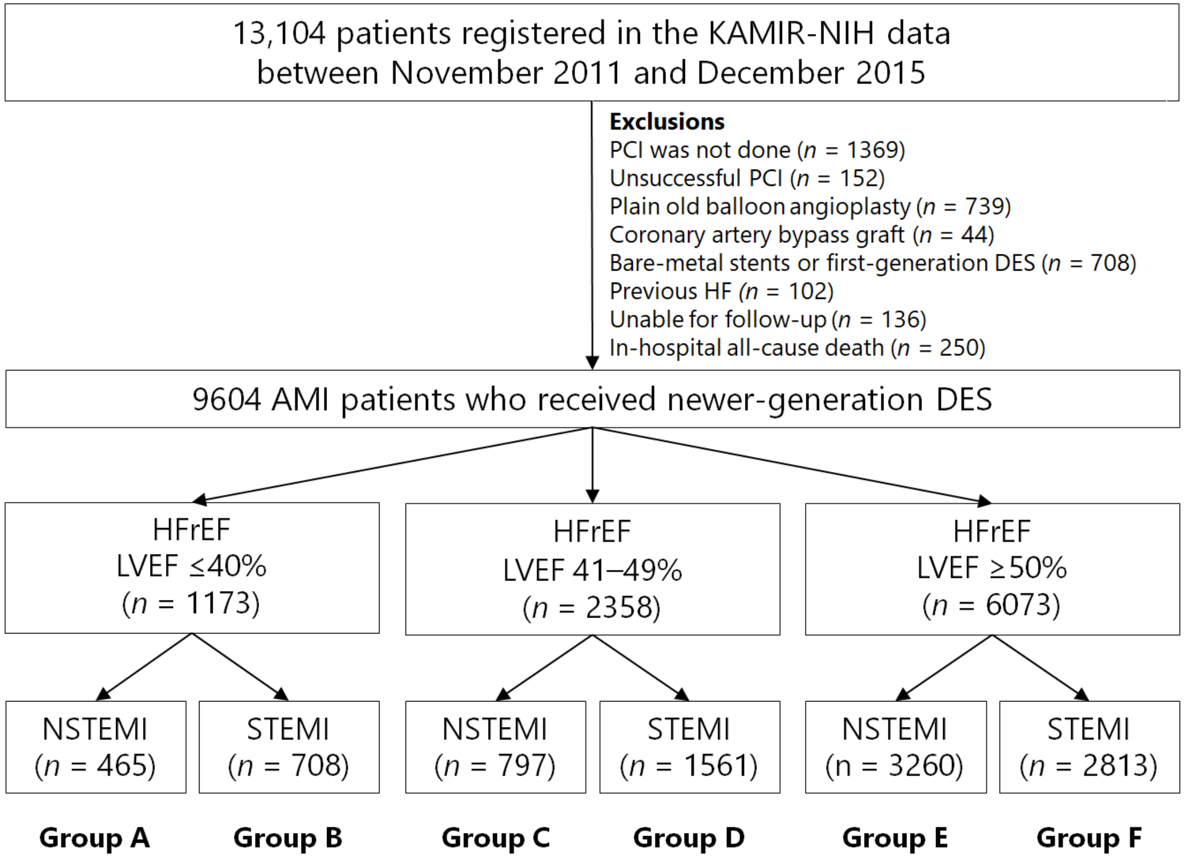

Figure S1. Flowchart after exclusion of in-hospital mortality. KAMIR-NIH, Korea Acute Myocardial Infarction Registry-National Institute of Health; PCI, percutaneous coronary intervention; DES, drug-eluting stent; HFrEF, heart failure with reduced ejection fraction; HFmrEF, heart failure with mildly reduced ejection fraction; HFpEF, heart failure with preserved ejection fraction; LVEF, left ventricular ejection fraction; NSTEMI, non-ST-segment elevation MI; STEMI, ST-segment elevation MI.

Figure S2. Kaplan-Meier analysis of POCO (A), all-cause death (B), cardiac death (C), and non-cardiac death (D) during a 3-year follow-up period after excluding in-hospital mortality.

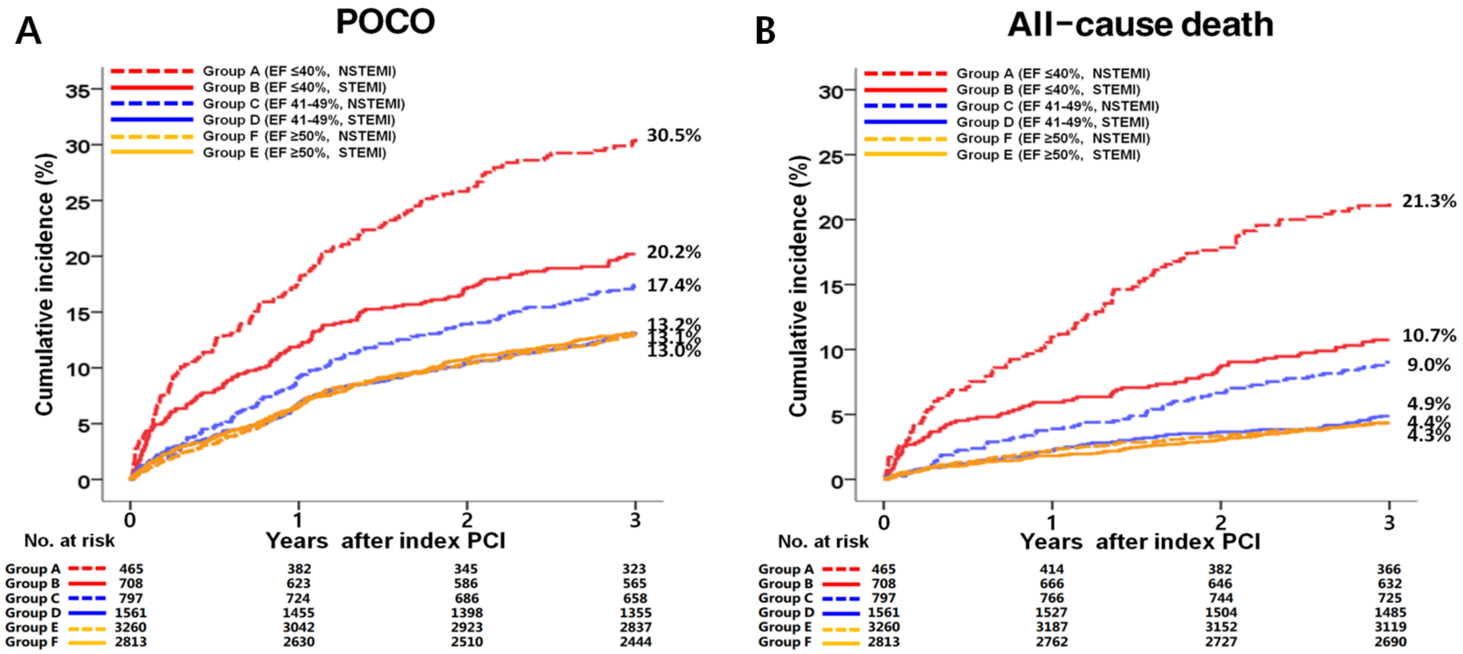

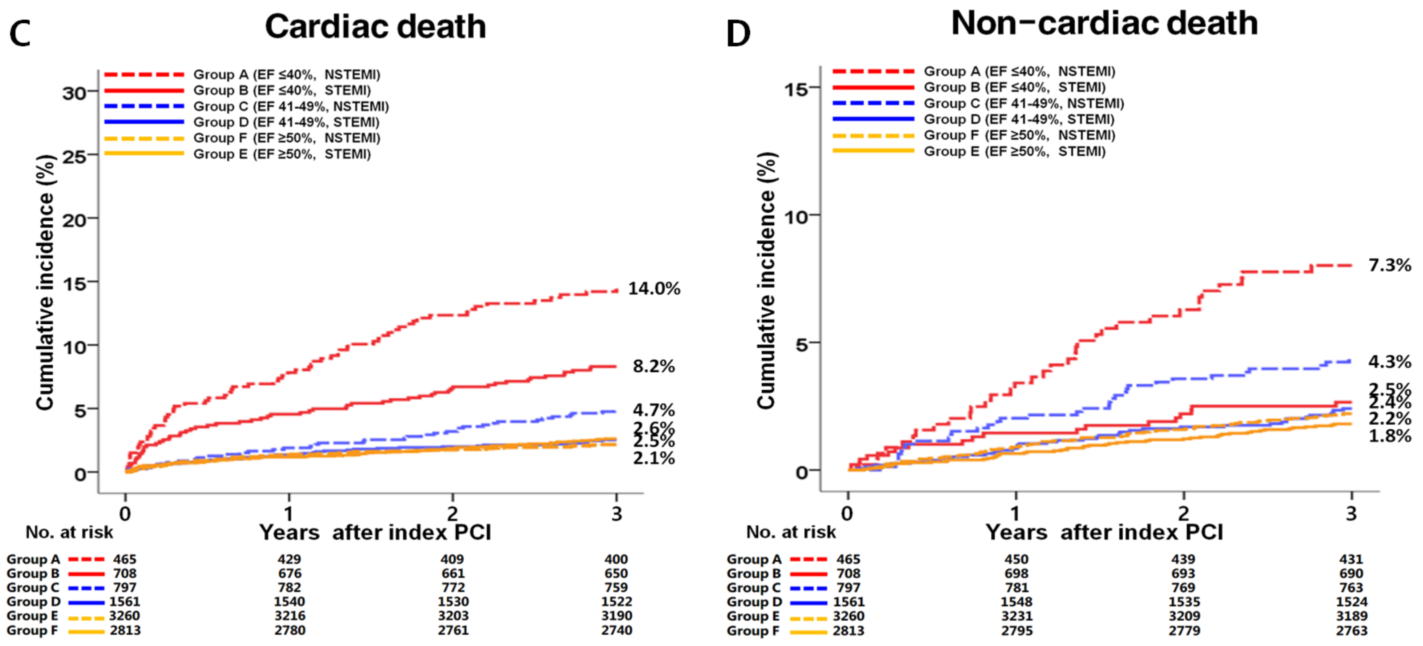
C
Cardiac death
Group A (EF ≤40%, NSTEMI)
Group B (EF ≤40%, STEMI)
Group C (EF 41-49%, NSTEMI)
Group D (EF 41-49%, STEMI)
Group F (EF ≥50%, NSTEMI)
Group E (EF ≥50%, STEMI)
Cumulative incidence (%)
0
5
10
15
20
25
30
14.0%
8.2%
4.7%
2.6%
2.5%
2.1%
0
1
2
3
Years after index PCI
No. at risk
Group A 465 429 409 400
Group B 708 676 661 650
Group C 797 782 772 759
Group D 1561 1540 1530 1522
Group E 3260 3216 3203 3190
Group F 2813 2780 2761 2740
D
Non-cardiac death
Group A (EF ≤40%, NSTEMI)
Group B (EF ≤40%, STEMI)
Group C (EF 41-49%, NSTEMI)
Group D (EF 41-49%, STEMI)
Group F (EF ≥50%, NSTEMI)
Group E (EF ≥50%, STEMI)
Cumulative incidence (%)
0
5
10
15
7.3%
4.3%
2.5%
2.4%
2.2%
1.8%
0
1
2
3
Years after index PCI
No. at risk
Group A 465 450 439 431
Group B 708 698 693 690
Group C 797 781 769 763
Group D 1561 1548 1535 1524
Group E 3260 3231 3209 3189
Group F 2813 2795 2779 2763

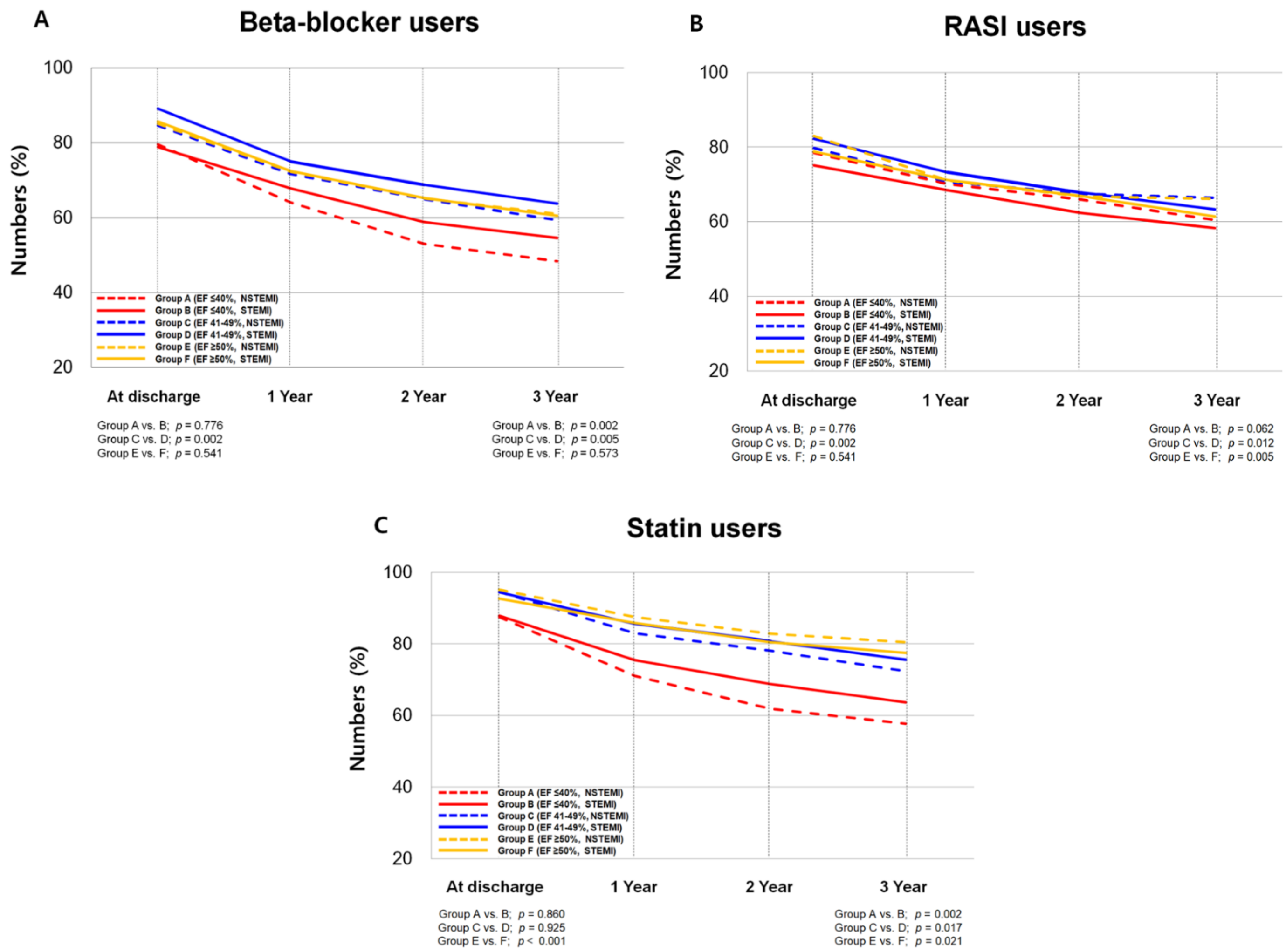

Figure S3. Trends in the use of beta-blockers, RASI, and statin users during the 3-year follow-up period after discharge. EF, ejection fraction; NSTEMI, non-ST-segment elevation myocardial infarction; STEMI, ST-segment elevation myocardial infarction; RASI, renin-angiotensin system inhibitor.

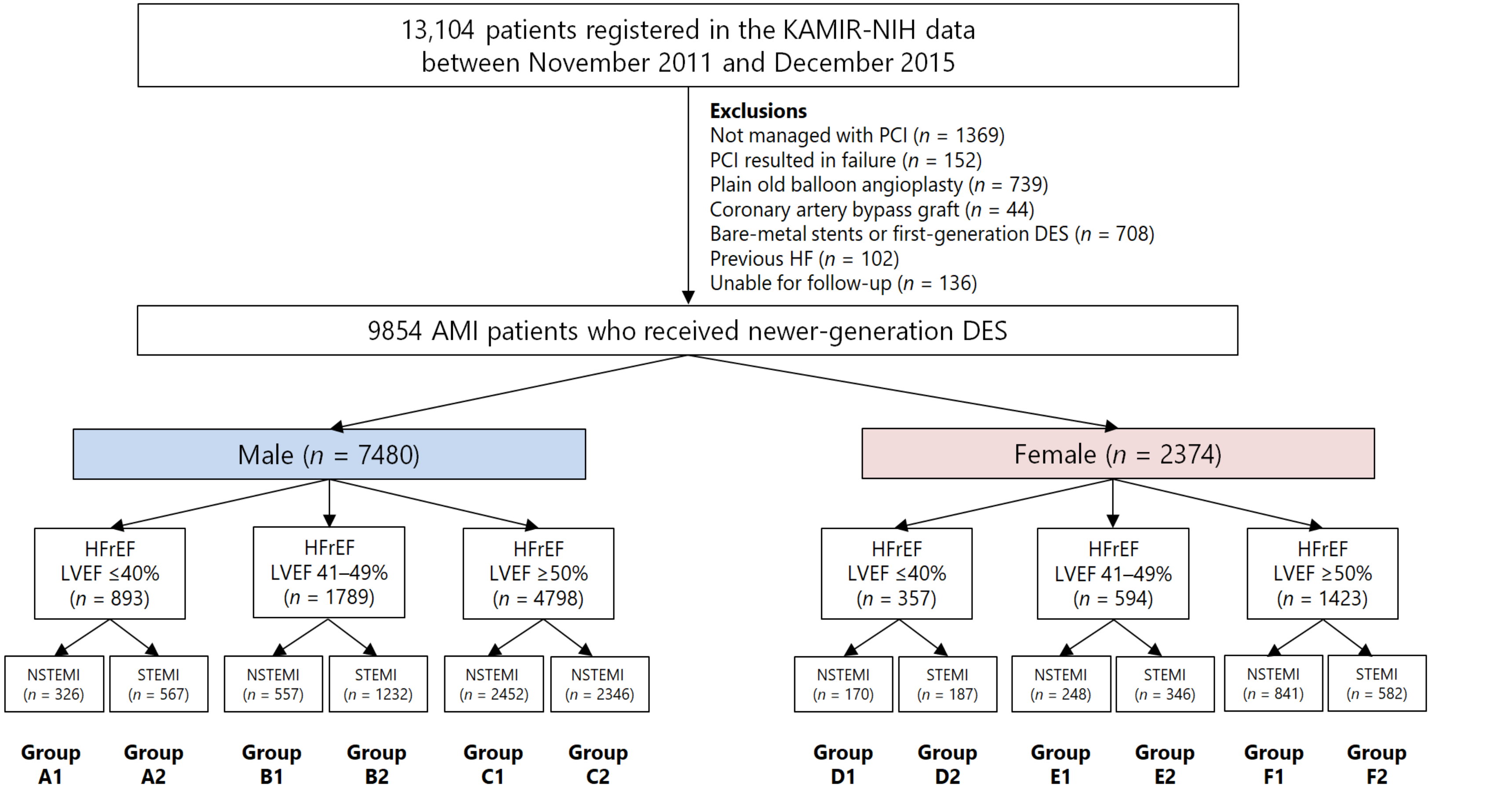

Figure S4. Flowchart based on male and female groups. KAMIR-NIH, Korea Acute Myocardial Infarction Registry-National Institute of Health; PCI, percutaneous coronary intervention; DES, drug-eluting stent; HFrEF, heart failure with reduced ejection fraction; HFmrEF, heart failure with mildly reduced ejection fraction; HFpEF, heart failure with preserved ejection fraction; LVEF, left ventricular ejection fraction; NSTEMI, non-ST-segment elevation MI; STEMI, ST-segment elevation MI.

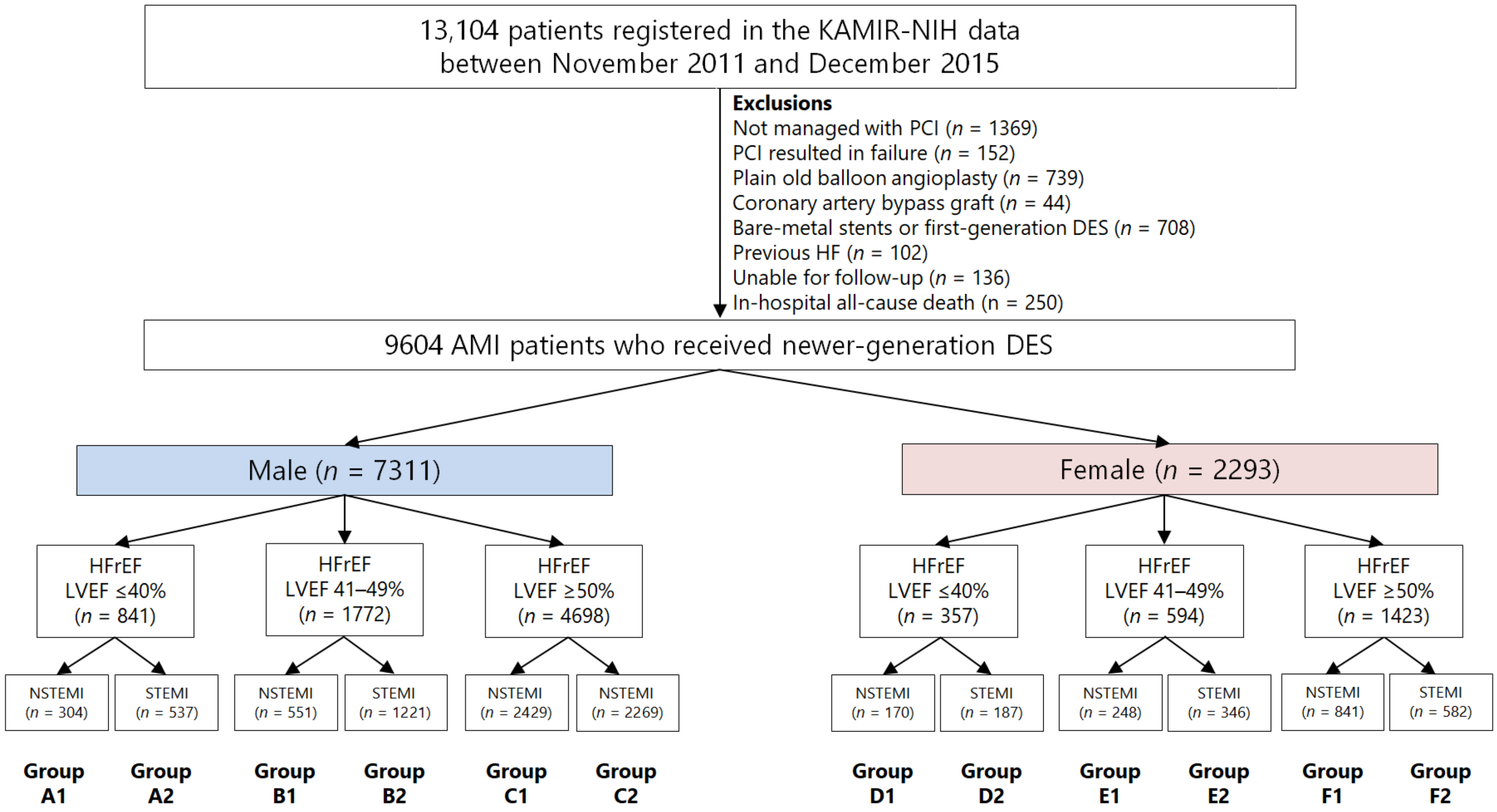

Figure S5. Flowchart based on male and female groups after exclusion of in-hospital mortality. KAMIR-NIH, Korea Acute Myocardial Infarction Registry-National Institute of Health; PCI, percutaneous coronary intervention; DES, drug-eluting stent; HFrEF, heart failure with reduced ejection fraction; HFmrEF, heart failure with mildly reduced ejection fraction; HFpEF, heart failure with preserved ejection fraction; LVEF, left ventricular ejection fraction; NSTEMI, non-ST-segment elevation MI; STEMI, ST-segment elevation MI.
